# PSIA: A Comprehensive Knowledgebase of Plant Self-incompatibility

**DOI:** 10.1093/gpbjnl/qzaf046

**Published:** 2025-05-21

**Authors:** Chen Wang, Hong Zhao, Hongkui Zhang, Sijie Sun, Yongbiao Xue

**Affiliations:** National Genomics Data Center, China National Center for Bioinformation, Beijing 100101, China; Beijing Institute of Genomics, Chinese Academy of Sciences, Beijing 100101, China; University of Chinese Academy of Sciences, Beijing 100049, China; Institute of Genetics and Developmental Biology, Chinese Academy of Sciences, Beijing 100101, China; National Genomics Data Center, China National Center for Bioinformation, Beijing 100101, China; Beijing Institute of Genomics, Chinese Academy of Sciences, Beijing 100101, China; University of Chinese Academy of Sciences, Beijing 100049, China; National Genomics Data Center, China National Center for Bioinformation, Beijing 100101, China; Beijing Institute of Genomics, Chinese Academy of Sciences, Beijing 100101, China; University of Chinese Academy of Sciences, Beijing 100049, China; National Genomics Data Center, China National Center for Bioinformation, Beijing 100101, China; Beijing Institute of Genomics, Chinese Academy of Sciences, Beijing 100101, China; University of Chinese Academy of Sciences, Beijing 100049, China; Institute of Genetics and Developmental Biology, Chinese Academy of Sciences, Beijing 100101, China

**Keywords:** Plant Self-Incompatibility Atlas, Genome, SI type, *S* gene, Evolution

## Abstract

Self-incompatibility (SI) is an important genetic mechanism in angiosperms that prevents inbreeding and promotes outcrossing, with significant implications for crop breeding, including genetic diversity, hybrid seed production, and yield optimization. In eudicots, SI is typically governed by a single *S*-locus containing tightly linked pistil and pollen *S*-determinant genes. Despite major advances in SI research, a centralized, comprehensive resource for SI-related genomic data remains lacking. To address this gap, we developed the Plant Self-Incompatibility Atlas (PSIA), a systematically curated knowledgebase providing an extensive compilation of plant SI, including genomic resources for SI species, *S* gene annotations, molecular mechanisms, phylogenetic relationships, and comparative genomic analyses. The current release of PSIA includes over 500 genome assemblies from 469 SI species. Using known *S* genes as queries, we manually identified and rigorously curated 3700 *S* genes. PSIA provides detailed *S*-locus information from assembled genomes of SI species and offers an interactive platform for browsing, BLAST searches, *S* gene analysis, and data retrieval. Additionally, PSIA serves as a unique platform for comparative genomic studies of *S*-loci, facilitating exploration of the dynamic processes underlying the origin, loss, and regain of SI. As a comprehensive and user-friendly resource, PSIA will greatly advance our understanding of angiosperm SI and serve as a valuable tool for crop breeding and hybrid seed production. PSIA is freely available at http://www.plantsi.cn.

## Graphical abstract

**Figure:**
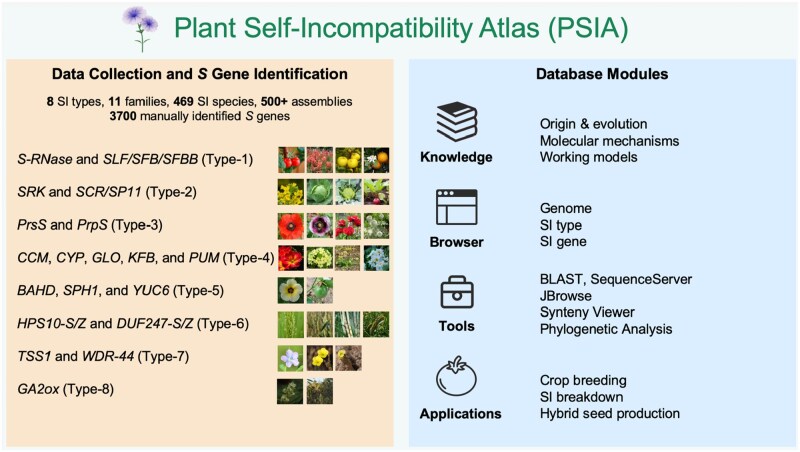

## Introduction

Flowering plants (angiosperms) comprise approximately 90% of all terrestrial plant species, forming the foundation of most ecosystems and playing a pivotal role in human livelihoods [1]. Self-incompatibility (SI), present in over 40% of angiosperms [2], is a reproductive mechanism that prevents self-fertilization in fertile plants. As a strict intraspecific barrier, SI prevents inbreeding and promotes outcrossing, which is essential for maintaining genetic diversity and the adaptive survival of populations [3]. While SI maintains genetic diversity in natural populations, it also presents challenges for breeding programs aiming for trait uniformity. Thus, understanding SI mechanisms is crucial for hybrid seed production and crop yield improvement, particularly in economically important genera such as *Solanum*, *Prunus*, *Citrus*, and *Brassica* [4].

In eudicots, SI is typically controlled by a highly polymorphic, multiallelic *S*-locus [5], whereas in grasses, it is governed by two independently inherited, polymorphic, multiallelic *S* and *Z* loci [6]. These loci encode distinct determinants that mediate pistil–pollen recognition and are inherited as a single segregating unit, with their variants termed *S* haplotypes [5]. In eudicots, self-pollen incompatibility (SPI), leading to self-pollen rejection, is triggered by the self-recognition of pistil and pollen *S* determinants encoded by the same *S* haplotype. In contrast, in grasses, SPI occurs only when both the *S* and *Z* haplotypes match. SI systems can be categorized into homomorphic (gametophytic and sporophytic) and heteromorphic (heterostyly) types based on their association with floral morphology [3,5,7–9]. Gametophytic self-incompatibility (GSI), which rejects self-pollen when its *S* haplotype matches either of the two *S* haplotypes in the pistil, has been identified in 17–25 plant families. In contrast, sporophytic self-incompatibility (SSI) inhibits self-pollen when the *S* haplotype of the sporophytic pollen parent matches either *S* haplotype in the diploid pistil. SSI is present in several plant families, including Brassicaceae, Asteraceae, Convolvulaceae, and Betulaceae [10–14]. Heterostyly, a floral polymorphism found in 28 families, including Primulaceae, Turneraceae, Linaceae, and Oleaceae [15–18], combines morphological and physiological incompatibility to prevent within-morph fertilization.

Given the substantial variation in the molecular mechanisms underlying SI systems across different plant families, these systems can be categorized into eight distinct types. The most widespread mechanism is gametophytic type-1 SI, governed by the pistil* S-RNase* and pollen* SLF.* This mechanism has been identified in Plantaginaceae [19–22], Solanaceae [23–25], Rosaceae [26–28], and Rutaceae [29]. Sporophytic type-2 SI, characteristic of Brassicaceae, is determined by *SRK* and *SP11*/*SCR* [10,11]. Gametophytic type-3 SI, observed in *Papaver rhoeas*, is controlled by *PrsS* and *PrpS* [30,31]. Heterostyly involves a hemizygous *S*-locus that regulates both floral morphology and incompatibility responses. This mechanism includes type-4 SI in Primulaceae, governed by *CYP*, *GLO2*, *KFB*, *CCM*, and *PUM* [15]; type-5 SI in Turneraceae, defined by *SPH1*, *YUC6*, and *BAHD* [16]; type-7 SI in Linaceae, determined by *TSS1* and *WDR-44* [17]; and type-8 SI in Oleaceae, governed by *GA2ox* [18]. Gametophytic type-6 SI in Poaceae is controlled by the *S* and *Z* loci, both of which encode HPS10 and DUF247I/II [6,32,33].

Advances in third-generation sequencing technologies have significantly improved plant genome assembly, leading to the development of dedicated genomic databases. For example, the Sol Genomics Network [34] serves as a comprehensive platform for Solanaceae species, integrating genomic, genetic, and breeding data, while the Genome Database for Rosaceae [35] provides a centralized repository for Rosaceae genomic and breeding resources. Despite these advancements, SI information remains largely overlooked in plant genomic studies and family-level databases, resulting in the underutilization of *S*-locus genomic resources. Notably, species within Solanaceae, Plantaginaceae, Rosaceae, and Rutaceae share the same SI type; however, no existing database integrates *S*-locus data across these families for systematic analysis. Given the increasing availability of sequenced plant genomes, a comprehensive SI knowledgebase is urgently needed to facilitate research on SI mechanisms, evolution, and applications in crop breeding.

To address this gap, we developed the Plant Self-Incompatibility Atlas (PSIA), a user-friendly and systematically curated database that integrates over 500 genome assemblies from 469 SI species across 11 plant families, covering all 8 SI types. PSIA consolidates extensive SI information, including its origin, evolution, molecular mechanisms, and working models. Additionally, we systematically identified and annotated 3700 *S* genes, with manual curation ensuring accuracy. PSIA provides a suite of analytical tools, including genome-wide BLAST, *S* gene-specific BLAST, genome browsing, and SI data analysis. To our knowledge, PSIA is the first knowledgebase to systematically compile and analyze SI-related genomic data across all plant species with well-characterized molecular mechanisms. Moreover, it is the first resource to integrate information on SI origin, evolution, and molecular mechanisms at the angiosperm-wide level. Consequently, PSIA represents the most comprehensive SI knowledgebase to date, serving as an essential resource for researchers investigating plant reproductive biology, SI mechanisms, and their implications for plant breeding.

## Database construction and content

### Integration of genome resources

**Figure 1:**
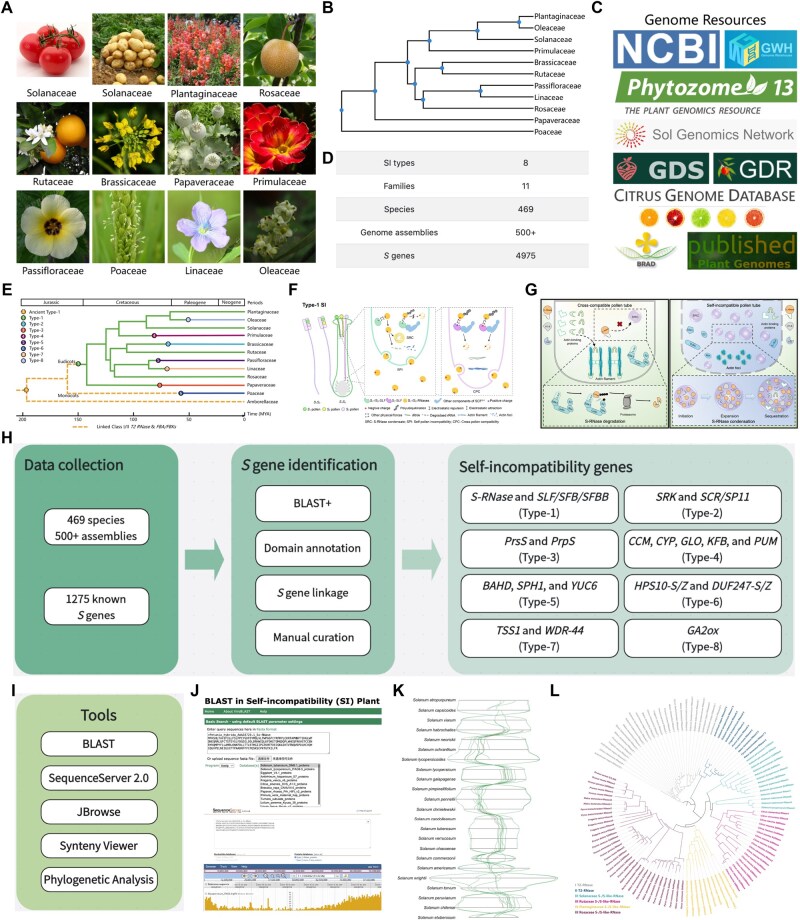
Overview of PSIA **A**. Representative species illustrating the eight known SI types. **B**. Phylogenetic relationships of all currently included plant families in PSIA. **C**. Available genome resources for SI species. **D**. Summary of SI-related data in the latest PSIA release. **E**. Evolutionary origins and diversification of the eight SI types. **F**. Schematic representation of the molecular mechanism underlying type-1 SI, alongside a comparative overview of all eight SI types in PSIA. **G**. Experimentally derived working model of SI in *Petunia hybrida*. **H**. Computational pipeline for *S* gene identification. **I**. Key analytical tools integrated into PSIA. **J**. Functional capabilities of the BLAST and JBrowse tools in PSIA. **K**. Example output of synteny analysis comparing *Solanum S*-loci. **L**. Phylogenetic reconstruction of S-RNases across representative families, including Plantaginaceae, Solanaceae, Rosaceae, and Rutaceae. PSIA, Plant Self-Incompatibility Atlas; SI, self-incompatibility.

Recent studies have identified 8 distinct molecular types of SI, spanning 11 families that include crops, fruits, ornamental plants, and model species (**Figure 1**A and B). To construct a comprehensive knowledgebase of SI, all publicly available genome assemblies for these families were retrieved from multiple databases, including National Center for Biotechnology Information (NCBI, https://www.ncbi.nlm.nih.gov/), Genome Warehouse (GWH, https://ngdc.cncb.ac.cn/gwh) [36], Published Plant Genomes (https://www.plabipd.de/plant_genomes_pa.ep), Phytozome (v13, https://phytozome-next.jgi.doe.gov) [37], Sol Genomics Network (https://solgenomics.net) [34], Genome Database for Rosaceae (GDR, https://www.rosaceae.org) [35], Genome Database for Strawberry (GDS, http://eplant.njau.edu.cn/strawberry/) [38], Citrus Genome Database (https://www.citrusgenomedb.org), Brassicaceae Database (BRAD, http://www.brassicadb.cn) [39], and other online resources such as Figshare and Google Drive (Figure 1C). The current release of PSIA includes over 500 genome assemblies from 469 SI species. To enable comprehensive retrieval of SI data, we implemented a fuzzy search function that accepts both scientific and common names (*e.g.*, “*Solanum lycopersicum*” and “tomato”, respectively). Figure 1D and Figure S1 provide an overview of the SI data available in PSIA, while Figure S2 displays the species tree of the eight SI types included in the database.

### Knowledge of SI origin, evolution, molecular mechanisms, and working models

To facilitate systematic SI research, PSIA incorporates comprehensive information on plant SI, including its origin, evolution, molecular mechanisms, and reported working models. Among eudicots, type-1 SI is the most ancient and widely distributed, while types 2–8 exhibit family-specific emergence. Phylogenomic and genetic analyses by Zhao et al. [40] suggest that *S-RNase*, *SLF*/*SFB*/*SFBB*, and type-1 *S*-loci trace back to the most recent common ancestor (MRCA) of eudicots. Under selective pressure favoring outcrossing, type-1 SI has been preserved in Plantaginaceae, Solanaceae, Rosaceae, and Rutaceae through selective deletion or inactivation of duplicate *S*-loci following whole-genome duplication (WGD) [40]. In contrast, Brassicaceae, Papaveraceae, Primulaceae, Turneraceae, Linaceae, and Oleaceae have lost type-1 SI via *S*-locus deletions or the maintenance of duplications, giving rise to new, family-specific SI systems (types 2–5, 7, and 8) (Figure 1E). Given that the ancient type-1 *S*-locus, encoding Class I/II T2 RNase and FBA/FBK proteins, originated from the MRCA of angiosperms, it is plausible that monocots acquired type-6 SI after the loss of type-1 SI (Figure 1E, Figure S3). To enhance understanding, PSIA provides molecular mechanism summaries for all eight SI types through schematic illustrations (Figure 1F, Figures S4–S10). Additionally, PSIA includes two experimentally derived working models of *Petunia hybrida* SI (Figure 1G) [41,42], providing the theoretical models for type-1 SI research.

### *S* gene identification and curation

The *S*-loci from assembled genomes of SI species were comprehensively analyzed in PSIA (Figure 1H) based on well-characterized types of molecular mechanisms. First, we retrieved all publicly available *S* gene sequences from the NCBI Nucleotide and Protein databases, compiling a dataset of 1275 nucleotide sequences and 1130 protein sequences. Using BLAST+ [43] with default parameters, we identified *S* gene candidates across the collected genome assemblies. Putative *S* gene coding regions were manually curated to ensure accurate splice site annotations. To refine candidate selection, we applied multiple filtering criteria, including the presence of conserved domains and genomic linkage distances. Specifically, type-1 *SLF*/*SFB*/*SFBB* genes were identified based on the presence of F-box and FBA/FBK domains, type-1 *S-RNase* genes were characterized by the Ribonuclease T2 family domain, and type-6 *DUF247I*/*II-S*/*Z* genes were distinguished by the DUF247 domain. Additionally, genomic linkage distances were incorporated as an additional filtering criterion, as *S* genes within each SI family exhibit characteristic genomic distributions, with linked male and female determinant *S* genes typically falling within a similar genomic span. To enhance candidate selection accuracy, we compared these distances with those reported in previous SI studies. For example, the *S*-locus spans approximately 17 Mb in *S. lycopersicum* (Solanaceae) [44], 1.2 Mb in *Antirrhinum* species (Plantaginaceae) [45], 1.36–2.64 Mb in *Pyrus* and *Malus* species (Rosaceae) [46], and 198–378 kb in *Citrus* species (Rutaceae) [29]. Based on the aforementioned strategies, we identified and manually curated a total of 3700 *S* genes in PSIA.

### Useful tools

PSIA integrates user-friendly tools, including BLAST, SequenceServer, JBrowse, Synteny Viewer, and Phylogenetic Analysis (Figures 1I–L). These tools can be accessed through the navigation bar on the database interface (Figure S11). Sequence similarity analysis is a fundamental bioinformatics method for detecting sequence homology and inferring potential functional relationships among genes. While the NCBI BLAST database remains a standard tool, its utility in SI research is constrained. To overcome these limitations, PSIA integrates both ViroBLAST [47] and SequenceServer [48] platforms, providing specialized analytical capabilities for SI studies including genome-wide and *S* gene-specific BLAST. Local genome-wide BLAST databases have been established using several genome assemblies representing different SI types. The BLAST procedure is straightforward, and optimal alignment results can be obtained using default parameters (Figure S12). PSIA hosts the most comprehensive collection of *S* gene sequences related to plant SI. To facilitate the utilization of these *S* gene resources, local databases of SequenceServer have been developed using thousands of *S* genes identified and curated by PSIA. SequenceServer enables faster and more accurate identification of *S* genes, with a user-friendly workflow (Figure S13), which is expected to significantly enhance the efficiency of SI research.

Genome browsers implemented on servers enable users to visualize genomic data online. For genome assemblies with accessible GFF files, a JBrowse function [49] has been provided, enabling researchers to browse the entire genome, *S*-locus regions, and *S* gene annotations (Figure S14). The SynVisio component (https://github.com/kiranbandi/synvisio) facilitates comparative genomic studies of different genome assemblies and *S*-loci. Synteny analysis of the *S*-loci will contribute to a deeper understanding of the dynamic evolution of SI (Figures S15 and S16). Additionally, phylogenetic trees have been constructed using IQ-TREE [50] based on the *S* genes identified in this study, which will aid in investigating their origins and relationships (Figure S17).

### Implementation

PSIA runs on a Linux-based Apache (https://httpd.apache.org) web server using PHP (a popular general-purpose scripting language, especially suited to web development; https://www.php.net) as the backend. The web interface is built with Bootstrap 5 (a powerful, extensible, and feature-packed frontend toolkit; https://getbootstrap.com), HTML5, CSS, and JavaScript. MySQL (http://www.mysql.org) has been adopted as the relational database system for information storage. All code is developed using Visual Studio (https://visualstudio.microsoft.com), a powerful and versatile integrated development environment (IDE) for developers and teams. PSIA is hosted on the Elastic Cloud Server (ECS) of HUAWEI CLOUD (https://activity.huaweicloud.com) to provide stable web services. The website has been tested to ensure functionality across various operating systems and web browsers, including Google Chrome, Firefox, and Microsoft Edge.

## Case study

PSIA integrates genome assemblies from species with well-characterized SI molecular mechanisms, enabling systematic identification and analysis of *S* genes. To demonstrate its utility in exploring *S*-locus information through high-quality genomic assemblies, we present a case study of *S. lycopersicum*, a representative species of the Solanaceae family. The PSIA interface provides access to SI type classifications and *S* gene information for Solanaceae via the “Browse” section. Users can navigate to the “Species” section for a direct link to *S. lycopersicum* or utilize a fuzzy search (*e.g.*, inputting “tomato”) to retrieve relevant entries, including wild tomato species (Figure S11A).

**Figure 2:**
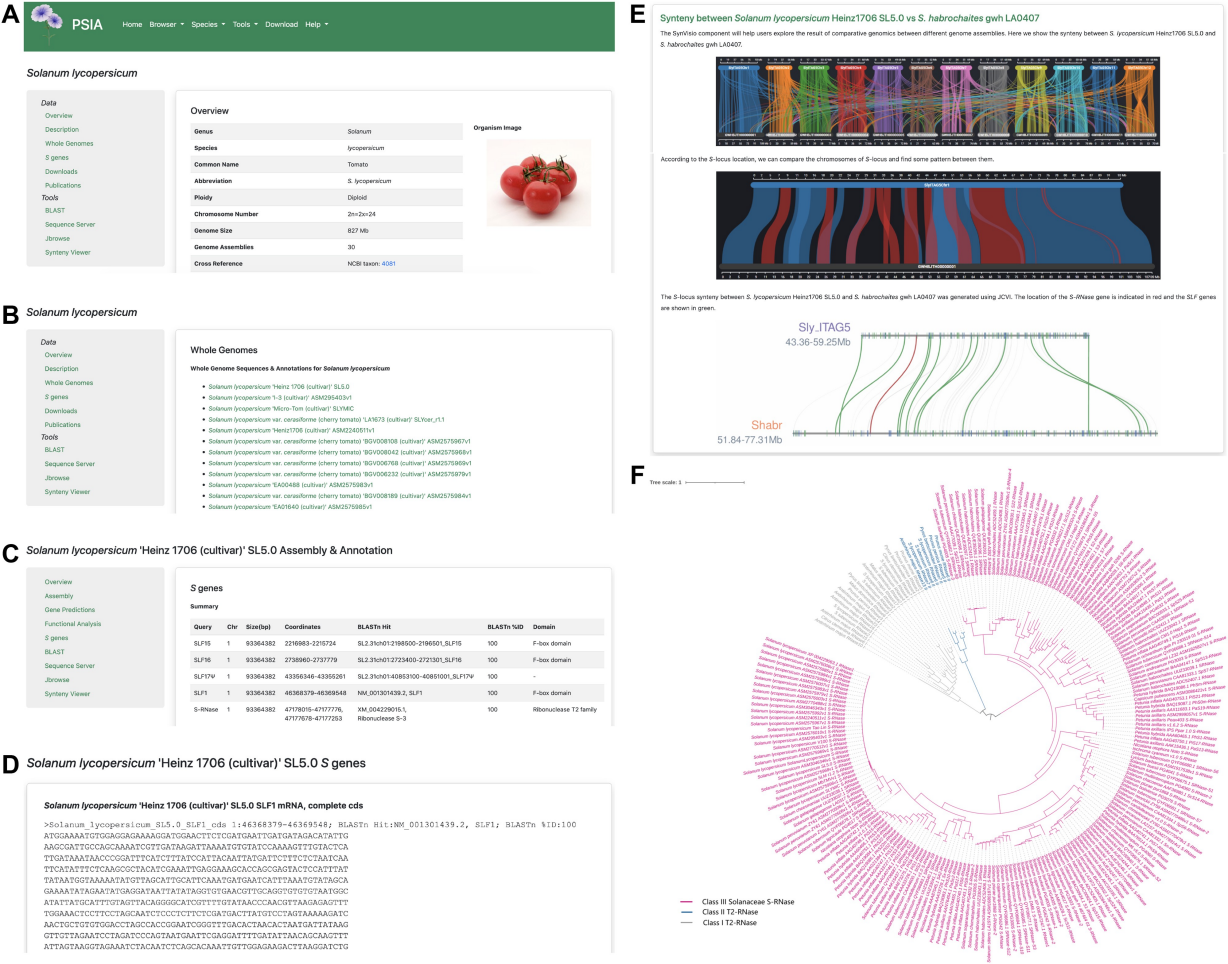
SI information and analysis of *Solanum lycopersicum* **A**. Species page of *S. lycopersicum*. **B**. Genome assembly list of *S. lycopersicum*. **C**. Summary of *S* genes in *S. lycopersicum* Heinz 1706 SL5.0 assembly. **D**. Sequence of the *SLF1* gene in the *S. lycopersicum* Heinz 1706 SL5.0 assembly. **E**. Synteny analysis output between *S. lycopersicum* Heinz 1706 SL5.0 and *S. habrochaites* gwh LA0407. **F**. Phylogenetic analysis output of S-RNases in Solanaceae.

The *S. lycopersicum* interface comprises two main sections: “Data” and “Tools”. The “Data” section includes: (1) Overview, summarizing species-specific details (**Figure 2**A); (2) Description, providing taxonomic and biological information; (3) Whole Genomes, listing 30 available genome assemblies (Figure 2B); (4) *S* genes, summarizing the annotation of identified *S* genes across these assemblies; (5) Downloads, offering *S* gene sequences from the 30 genomes; and (6) Publications, compiling relevant genomic literature. For example, selecting “Heinz 1706 (cultivar) SL5.0 assembly” under “Whole Genomes” redirects users to a dedicated page featuring: (1) Overview, summarizing assembly statistics; (2) Assembly, providing genome download links; (3) Gene Predictions, detailing structural annotations; (4) Functional Analysis, presenting gene function annotations; and (5) *S* genes, displaying sequence information specific to the selected assembly (Figure 2C and D).

PSIA’s tools enable in-depth analysis of *S*-loci and *S* genes in tomatoes. The genome-wide BLAST enables homology searches against local databases of *S. lycopersicum* genomes, coding sequences (CDS), and proteins. This eliminates the need for users to manually download genome data and execute BLAST+ commands via Linux. Instead, users can input a query sequence, select a species-specific database, and utilize optimized default parameters for homology searches (Figure S12). The integrated SequenceServer hosts a comprehensive repository of *S. lycopersicum SLF* and *S-RNas*e genes, significantly enhancing the accuracy and efficiency of *S* gene identification in newly assembled genomes of tomatoes (Figure S13). To further explore *S*-locus architecture, PSIA integrates JBrowse for visualizing *S*-locus genomic features and annotated *S* gene structures. For instance, using the *S* gene annotations identified in *S. lycopersicum* “Heinz 1706 (cultivar)” SL5.0 assembly (Figure 2C), users can visualize the complete gene structure and annotation details of *S-RNase* by entering genomic coordinates 1:47178015-47177253 in JBrowse (Figure S14). Comparative synteny analysis of the *S*-loci between cultivated tomato (*S. lycopersicum*) and wild tomato (*Solanum habrochaites*) revealed conserved collinearity as well as structural variations (Figure 2E, Figure S15). Notably, wild tomatoes retain functional SI systems that promote outcrossing and serve as reservoirs of novel alleles. Comprehensive characterization of *S* genes in wild and cultivated tomato species enables precise manipulation of cross-pollination barriers, thereby enhancing hybrid breeding efficiency. Extended synteny analysis across *Solanum* species (Figure S16) further elucidated *S*-locus diversification within the genus. To investigate the evolutionary trajectory of *S* genes within Solanaceae, we performed phylogenetic analyses of *S-RNase* and *SLF* genes across Solanaceae species (Figure 2F, Figure S18) and extended our analysis to four families exhibiting type-1 SI (Figures S19 and S20). These phylogenetic insights contribute to a broader understanding of type-1 SI systems.

## Discussion and conclusion

Research on angiosperm SI dates back to the 19th century. While substantial progress has been made, the molecular mechanisms underlying this complex reproductive system remain incompletely understood. SI research holds both fundamental biological significance and practical importance, particularly in crop breeding, quality improvement, and fruit production [4]. The availability of high-quality plant genomes has greatly facilitated the identification and analysis of *S* genes, especially in the most widespread type-1 SI system. Type-1 *S*-loci typically span large genomic regions containing a female determinant (*S-RNase*) and approximately 15–32 male determinants (*SLF*/*SFB*/*SFBB* genes) [29,44–46], necessitating chromosome-scale assemblies for comprehensive structural resolution. However, existing plant genomic databases largely overlook *S* gene annotation and analysis. For example, the Sol Genomics Network primarily focuses on limited Solanaceae species, emphasizing gene expression, quantitative trait loci (QTLs), and genetic markers [34]. To date, no database has systematically integrated available genome assemblies to comprehensively annotate and analyze *S* genes across taxa, representing a critical gap that hinders SI research.

The PSIA knowledgebase aims to provide the most comprehensive online platform for plant SI research by integrating genomic data, *S*-locus information, *S* gene sequences, and analytical tools. For researchers unfamiliar with SI systems, PSIA offers a systematic introduction to SI classification, origin, evolution, and experimentally derived working models. For SI specialists, the platform features a locally implemented BLAST service via SequenceServer, enabling rapid and accurate identification of *S* genes in newly assembled genomes using the most extensive collection of *S* gene sequences. Additionally, bioinformatics users can download *S* gene sequences for customized analyses. PSIA’s data integration spans angiosperm-wide comparisons, facilitating macroevolutionary investigations of SI dynamics. Notably, the four families exhibiting type-1 SI include numerous crops, fruits, and ornamental species. A comprehensive understanding of the molecular mechanisms and evolutionary trajectory of type-1 SI requires the systematic integration of *S* gene data from these four families. Moreover, the platform’s Synteny Viewer supports both interspecies (*e.g.*, cultivated *vs*. wild relatives) and genus-level comparisons, providing insights into the dynamic evolution of SI. PSIA’s Phylogenetic Analysis module incorporates the most extensive *S* gene sequence collection to date, significantly advancing evolutionary studies of these loci.

PSIA’s comprehensive annotation and analysis of *S* genes can enhance hybrid breeding in tomatoes. Wild tomato (*S. habrochaites*) possesses valuable traits, such as disease resistance, stress tolerance, and improved fruit quality, which breeders aim to transfer into cultivated tomato (*S. lycopersicum*). Interspecific hybridization between cultivated (self-compatible) and wild (self-incompatible) tomatoes requires a comprehensive understanding of *S* gene function to either maintain SI for breeding diversity or overcome SI for successful hybrid production. Manipulating *S* gene expression offers a viable approach to overcoming reproductive barriers and enabling gene flow between wild and cultivated species. For example, disrupting or silencing *S* genes using CRISPR-Cas9 or RNA interference can restore self-compatibility in hybrids, thereby improving seed production efficiency. Additionally, synteny analysis between cultivated and wild tomatoes enables researchers to track evolutionary changes in reproductive mechanisms, providing insights into how domestication has influenced SI systems in tomatoes.

PSIA has significant potential for further advancements, and we plan to continuously update and expand its functionality. For instance, the database will periodically incorporate genomic and *S*-locus information from representative species across the 11 families with well-characterized molecular mechanisms. Furthermore, as novel molecular mechanisms of SI are discovered in other plant families, PSIA will systematically integrate these new findings, providing a continuously evolving and comprehensive knowledgebase. Given the tissue-specific expression patterns of pistil and pollen *S* genes, future versions will integrate relevant transcriptome data to enable *S* gene expression analysis. Additional functionalities, such as primer-design tools, will be implemented based on user needs, broadening the platform’s applicability in functional genomics and breeding research. These planned updates will ensure that PSIA remains a comprehensive and up-to-date resource for SI research.

In conclusion, PSIA represents a critical advancement in plant SI research, leveraging high-quality genome assemblies to systematically explore *S*-locus information and addressing the current limitations in SI-related genomic databases. Looking ahead, regular updates incorporating newly sequenced genomes and the latest insights into SI molecular mechanisms will enhance its utility as a continuously evolving resource. By providing a comprehensive, user-friendly platform, PSIA will facilitate novel discoveries in plant reproductive biology, improve our understanding of *S*-locus evolution, and support applied research in crop breeding and quality improvement.

## Supplementary Material

qzaf046_Supplementary_Data

## Data Availability

PSIA is freely available at http://www.plantsi.cn. PSIA has been submitted to Database Commons [51] at the National Genomics Data Center (NGDC), China National Center for Bioinformation (CNCB), which is publicly accessible at https://ngdc.cncb.ac.cn/databasecommons/database/id/8169.

## CRediT author statement

**Chen Wang:** Conceptualization, Data curation, Formal analysis, Investigation, Methodology, Visualization, Software, Writing – original draft, Writing – review & editing. **Hong Zhao:** Conceptualization, Funding acquisition, Methodology, Visualization, Writing – original draft, Writing – review & editing. **Hongkui Zhang:** Data curation, Formal analysis. **Sijie Sun:** Data curation, Formal analysis. **Yongbiao Xue:** Conceptualization, Funding acquisition, Project administration, Supervision, Writing – original draft, Writing – review & editing. All authors have read and approved the final manuscript.
