# Supplementary material for: PSIA: A Comprehensive Knowledgebase of Plant Self-incompatibility
Source: Genomics Proteomics Bioinformatics. 2025 May 21;23(3):qzaf046. doi: 10.1093/gpbjnl/qzaf046 (PMC12396629; doi:10.1093/gpbjnl/qzaf046)
Supplement: qzaf046_Supplementary_Data [file qzaf046_supplementary_data.zip › FigureS11.pdf]

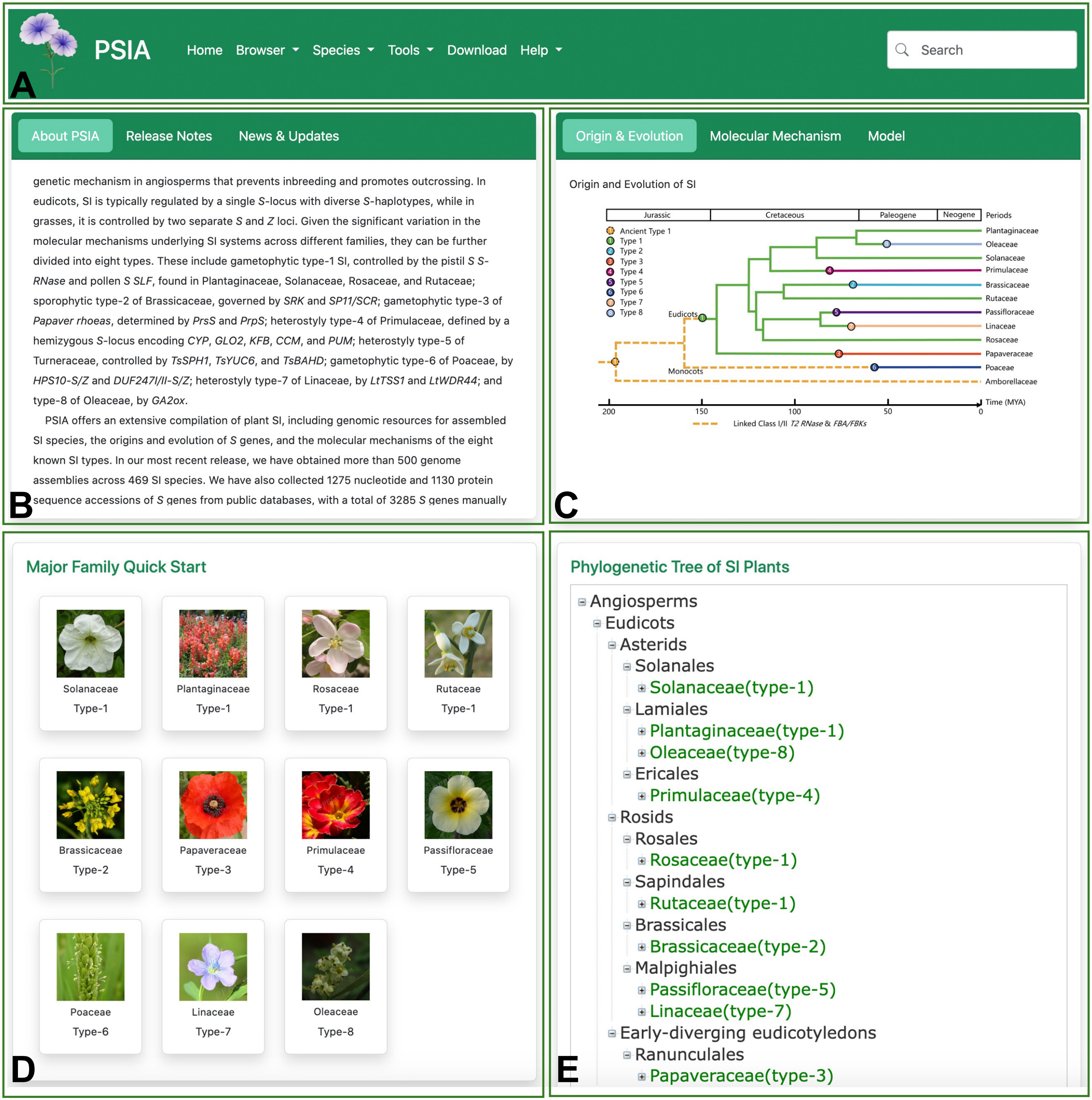
PSIA
Home
Browser
Species
Tools
Download
Help
Search
A
About PSIA
Release Notes
News & Updates
genetic mechanism in angiosperms that prevents inbreeding and promotes outcrossing. In eudicots, SI is typically regulated by a single S-locus with diverse S-haplotypes, while in grasses, it is controlled by two separate S and Z loci. Given the significant variation in the molecular mechanisms underlying SI systems across different families, they can be further divided into eight types. These include gametophytic type-1 SI, controlled by the pistil S S-RNase and pollen S SLF, found in Plantaginaceae, Solanaceae, Rosaceae, and Rutaceae; sporophytic type-2 of Brassicaceae, governed by SRK and SP11/SCR; gametophytic type-3 of Papaver rhoeas, determined by PrsS and PrpS; heterostyly type-4 of Primulaceae, defined by a hemizygous S-locus encoding CYP, GLO2, KFB, CCM, and PUM; heterostyly type-5 of Turneraceae, controlled by TsSPH1, TsYUC6, and TsBAHD; gametophytic type-6 of Poaceae, by HPS10-S/Z and DUF247I/II-S/Z; heterostyly type-7 of Linaceae, by LtTSS1 and LtWDR44; and type-8 of Oleaceae, by GA2ox.
PSIA offers an extensive compilation of plant SI, including genomic resources for assembled SI species, the origins and evolution of S genes, and the molecular mechanisms of the eight known SI types. In our most recent release, we have obtained more than 500 genome assemblies across 469 SI species. We have also collected 1275 nucleotide and 1130 protein sequence accessions of S genes from public databases, with a total of 3285 S genes manually
B
Origin & Evolution
Molecular Mechanism
Model
Origin and Evolution of SI
Jurassic
Cretaceous
Paleogene
Neogene
Periods
Ancient Type 1
Type 1
Type 2
Type 3
Type 4
Type 5
Type 6
Type 7
Type 8
Eudicots
Monocots
Plantaginaceae
Oleaceae
Solanaceae
Primulaceae
Brassicaceae
Rutaceae
Passifloraceae
Linaceae
Rosaceae
Papaveraceae
Poaceae
Amborellaceae
200
150
100
50
0
Time (MYA)
Linked Class I/II T2 RNase & FBA/FBKs
C
Major Family Quick Start
Solanaceae
Type-1
Plantaginaceae
Type-1
Rosaceae
Type-1
Rutaceae
Type-1
Brassicaceae
Type-2
Papaveraceae
Type-3
Primulaceae
Type-4
Passifloraceae
Type-5
Poaceae
Type-6
Linaceae
Type-7
Oleaceae
Type-8
D
Phylogenetic Tree of SI Plants
Angiosperms
Eudicots
Asterids
Solanales
Solanaceae(type-1)
Lamiales
Plantaginaceae(type-1)
Oleaceae(type-8)
Ericales
Primulaceae(type-4)
Rosids
Rosales
Rosaceae(type-1)
Sapindales
Rutaceae(type-1)
Brassicales
Brassicaceae(type-2)
Malpighiales
Passifloraceae(type-5)
Linaceae(type-7)
Early-diverging eudicotyledons
Ranunculales
Papaveraceae(type-3)
E
