# Supplementary material for: PSIA: A Comprehensive Knowledgebase of Plant Self-incompatibility
Source: Genomics Proteomics Bioinformatics. 2025 May 21;23(3):qzaf046. doi: 10.1093/gpbjnl/qzaf046 (PMC12396629; doi:10.1093/gpbjnl/qzaf046)
Supplement: qzaf046_Supplementary_Data [file qzaf046_supplementary_data.zip › FigureS12.pdf]

BLAST in Self-incompatibility (SI) Plant

Home About ViroBLAST Help

Basic Search - using default BLAST parameter settings

(1) Enter query sequences here in Fasta format

```
>Petunia_hybrida_AAA33729.1_Sx-RNase
MFKSHLTAVIFILLFSLPPIYGDFDYMQLVLTWPASFCYRPRYLCKRTAPNNFTIHGLWP
DNEQRRLQFCTSTEYSLFDGDILDDLDRHWIQLKFDKETGMQDQPLWHEQFRKHGTCCEN
RYKQMPYFLLAMRLKNKFDLLTTLRTHGIIPGTKHTFDEIQKAIKTVTNQVDPDLKCVQH
IQGVPELNEIGICFTPAADRFFPCPQSKSCPKTGTKILFR
```

Or upload sequence fasta file: 选择文件 未选择任何文件

(2) Program blastp

(3) Database(s)

- Solanum_tuberosum_DM8.1_proteins
- Solanum_lycopersicum_ITAG5.0_proteins
- Eggplant_V4.1_proteins
- Antirrhinum_hispanicum_S7_proteins
- Fragaria_vesca_v6_proteins
- Citrus_sinensis_DVS_A1.0_proteins
- Brassica_rapa_ChiifuV4.0_proteins
- Papaver_rhoeas_Prh_HiFi_v2_proteins
- Primula_veris_maternal_hap_proteins
- Turnera_subulata_proteins
- Lolium_perenne_Kyuss_39_proteins
- Linum_tenue_thrum_v1_proteins

And/or upload sequence fasta file: 选择文件 未选择任何文件

(4) Basic search Reset

Advanced Search - setting your favorite parameters below

(5)

Expect threshold	10
Word size	3
Max target sequences	50
Matrix	BLOSUM62
Gap costs	Existence: 11, Extension: 1
Filter	☐ Low complexity regions
Mask	☐ Mask for lookup table only ☐ Mask for lower case letters
Alignment	☐ Perform ungapped alignment
Alignment output format	pairwise
Other parameters	

Advanced search Reset

(6)

BLAST Result

Home About ViroBLAST Help

Inspect BLAST output

Filter current page by score:

Show - All - for each query sequence Filter

Re-parse current blast results (please select cutoff criterion):

◉ Similarity percentage Cutoff %: 95

○ Blast score Cutoff score: 1000 Parse again

Retrieve and download subject sequences in FASTA format: ◉ Entire sequence ○ Region mapped to query

☐ Check here to download All sequences... OR select particular sequences of interest below

Submit your selection of sequences to download

Query	Subject	Score	Identities (Query length)	Percentage	Expect
Petunia_hybrida_AAA33729.1_Sx-RNase	☐ DM8C01G14250.1	240	121/222 (220)	55	1e-80,
Petunia_hybrida_AAA33729.1_Sx-RNase	☐ DM8C05G04870.1	99.8	70/226 (220)	31	1e-25,
Petunia_hybrida_AAA33729.1_Sx-RNase	☐ DM8C05G04860.1	82.4	64/228 (220)	28	4e-19,
Petunia_hybrida_AAA33729.1_Sx-RNase	☐ DM8C05G03000.1	77.4	59/217 (220)	27	4e-17,
Petunia_hybrida_AAA33729.1_Sx-RNase	☐ DM8C04G00790.1	75.1	55/191 (220)	29	3e-16,
Petunia_hybrida_AAA33729.1_Sx-RNase	☐ DM8C04G00800.1	71.2	54/199 (220)	27	6e-15,
Petunia_hybrida_AAA33729.1_Sx-RNase	☐ DM8C05G02960.1	71.2	58/222 (220)	26	7e-15,
Petunia_hybrida_AAA33729.1_Sx-RNase	☐ DM8C09G14180.1	70.1	51/188 (220)	27	2e-14,
Petunia_hybrida_AAA33729.1_Sx-RNase	☐ DM8C07G02010.2	66.6	55/192 (220)	29	3e-13,
Petunia_hybrida_AAA33729.1_Sx-RNase	☐ DM8C07G02030.1	65.5	64/240 (220)	27	1e-12,
