# Supplementary material for: PSIA: A Comprehensive Knowledgebase of Plant Self-incompatibility
Source: Genomics Proteomics Bioinformatics. 2025 May 21;23(3):qzaf046. doi: 10.1093/gpbjnl/qzaf046 (PMC12396629; doi:10.1093/gpbjnl/qzaf046)
Supplement: qzaf046_Supplementary_Data [file qzaf046_supplementary_data.zip › FigureS13.pdf]

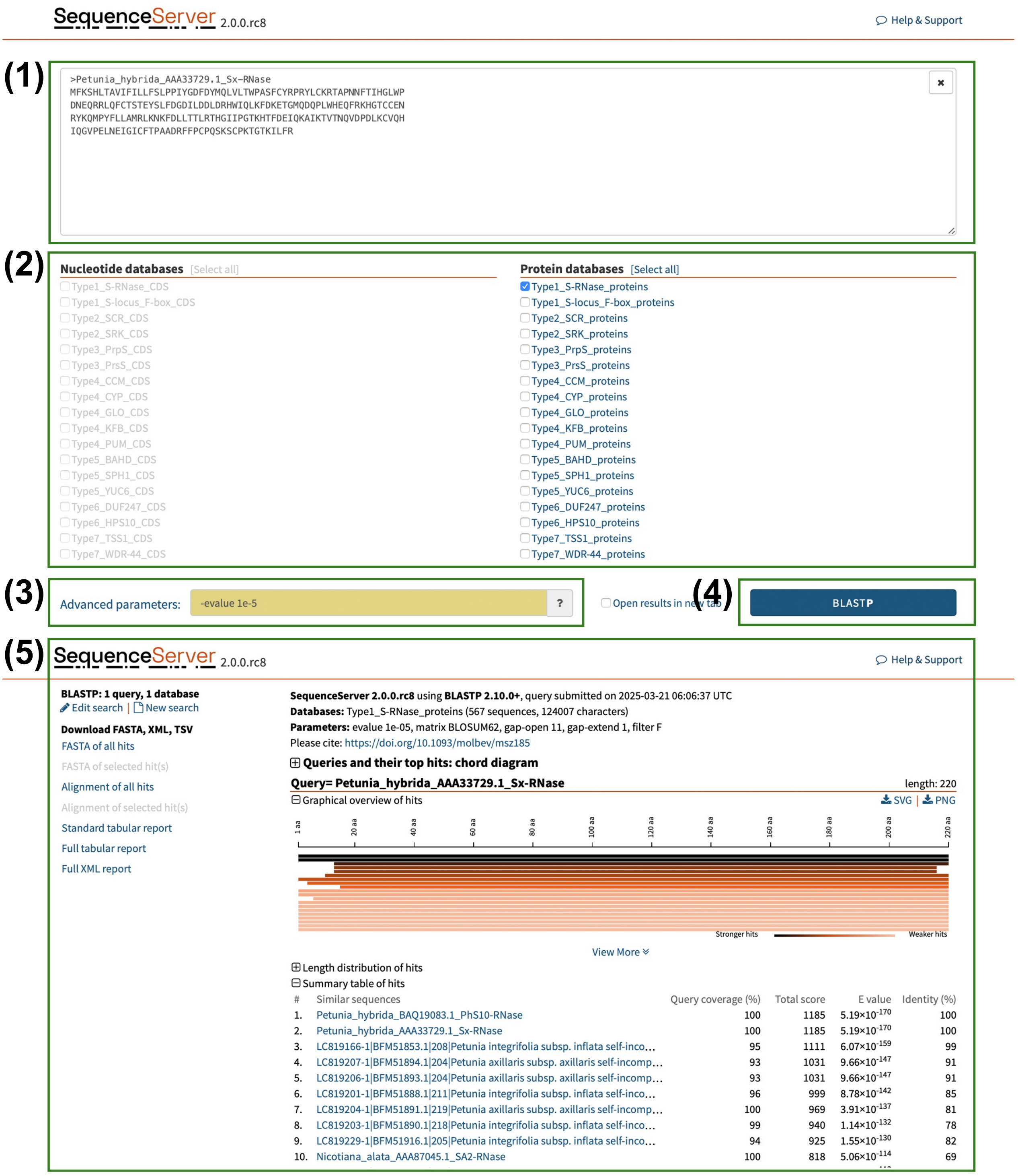

SequenceServer 2.0.0.rc8
Help & Support
(1)
>Petunia_hybrida_AAA33729.1_Sx-RNase
MFKSHLTAVIFILLFSLPPIYGDFDYMQLVLTWPASFCYRPRYLCKRTAPNNFTIHGLWP
DNEQRRLQFCTSTEYSLFDGDILDDLDRHWIQLKFDKETGMQDQPLWHEQFRKHGTCCEN
RYKQMPYFLLAMRLKNKFDLLTTLRTHGIIPGTKHTFDEIQKAIKTVTNQVDPDLKCVQH
IQGVPELNEIGICFTPAADRFFPCPQSKSCPKTGTKILFR
(2)
Nucleotide databases [Select all]
Type1_S-RNase_CDS
Type1_S-locus_F-box_CDS
Type2_SCR_CDS
Type2_SRK_CDS
Type3_PrpS_CDS
Type3_PrsS_CDS
Type4_CCM_CDS
Type4_CYP_CDS
Type4_GLO_CDS
Type4_KFB_CDS
Type4_PUM_CDS
Type5_BAHD_CDS
Type5_SPH1_CDS
Type5_YUC6_CDS
Type6_DUF247_CDS
Type6_HPS10_CDS
Type7_TSS1_CDS
Type7_WDR-44_CDS
Protein databases [Select all]
Type1_S-RNase_proteins
Type1_S-locus_F-box_proteins
Type2_SCR_proteins
Type2_SRK_proteins
Type3_PrpS_proteins
Type3_PrsS_proteins
Type4_CCM_proteins
Type4_CYP_proteins
Type4_GLO_proteins
Type4_KFB_proteins
Type4_PUM_proteins
Type5_BAHD_proteins
Type5_SPH1_proteins
Type5_YUC6_proteins
Type6_DUF247_proteins
Type6_HPS10_proteins
Type7_TSS1_proteins
Type7_WDR-44_proteins
(3)
Advanced parameters:
-evalue 1e-5
?
Open results in new tab
(4)
BLASTP
(5)
SequenceServer 2.0.0.rc8
Help & Support
BLASTP: 1 query, 1 database
Edit search | New search
Download FASTA, XML, TSV
FASTA of all hits
FASTA of selected hit(s)
Alignment of all hits
Alignment of selected hit(s)
Standard tabular report
Full tabular report
Full XML report
SequenceServer 2.0.0.rc8 using BLASTP 2.10.0+, query submitted on 2025-03-21 06:06:37 UTC
Databases: Type1_S-RNase_proteins (567 sequences, 124007 characters)
Parameters: evalue 1e-05, matrix BLOSUM62, gap-open 11, gap-extend 1, filter F
Please cite: https://doi.org/10.1093/molbev/msz185
Queries and their top hits: chord diagram
Query= Petunia_hybrida_AAA33729.1_Sx-RNase
length: 220
Graphical overview of hits
SVG | PNG
1 aa
20 aa
40 aa
60 aa
80 aa
100 aa
120 aa
140 aa
160 aa
180 aa
200 aa
220 aa
Stronger hits
Weaker hits
View More
Length distribution of hits
Summary table of hits
Similar sequences Query coverage (%) Total score E value Identity (%)
1. Petunia_hybrida_BAQ19083.1_PhS10-RNase 100 1185 5.19×10^-170 100
2. Petunia_hybrida_AAA33729.1_Sx-RNase 100 1185 5.19×10^-170 100
3. LC819166-1|BFM51853.1|208|Petunia integrifolia subsp. inflata self-inco… 95 1111 6.07×10^-159 99
4. LC819207-1|BFM51894.1|204|Petunia axillaris subsp. axillaris self-incomp… 93 1031 9.66×10^-147 91
5. LC819206-1|BFM51893.1|204|Petunia axillaris subsp. axillaris self-incomp… 93 1031 9.66×10^-147 91
6. LC819201-1|BFM51888.1|211|Petunia integrifolia subsp. inflata self-inco… 96 999 8.78×10^-142 85
7. LC819204-1|BFM51891.1|219|Petunia axillaris subsp. axillaris self-incomp… 100 969 3.91×10^-137 81
8. LC819203-1|BFM51890.1|218|Petunia integrifolia subsp. inflata self-inco… 99 940 1.14×10^-132 78
9. LC819229-1|BFM51916.1|205|Petunia integrifolia subsp. inflata self-inco… 94 925 1.55×10^-130 82
10. Nicotiana_alata_AAA87045.1_SA2-RNase 100 818 5.06×10^-114 69
