# Supplementary material for: PSIA: A Comprehensive Knowledgebase of Plant Self-incompatibility
Source: Genomics Proteomics Bioinformatics. 2025 May 21;23(3):qzaf046. doi: 10.1093/gpbjnl/qzaf046 (PMC12396629; doi:10.1093/gpbjnl/qzaf046)
Supplement: qzaf046_Supplementary_Data [file qzaf046_supplementary_data.zip › FigureS14.pdf]

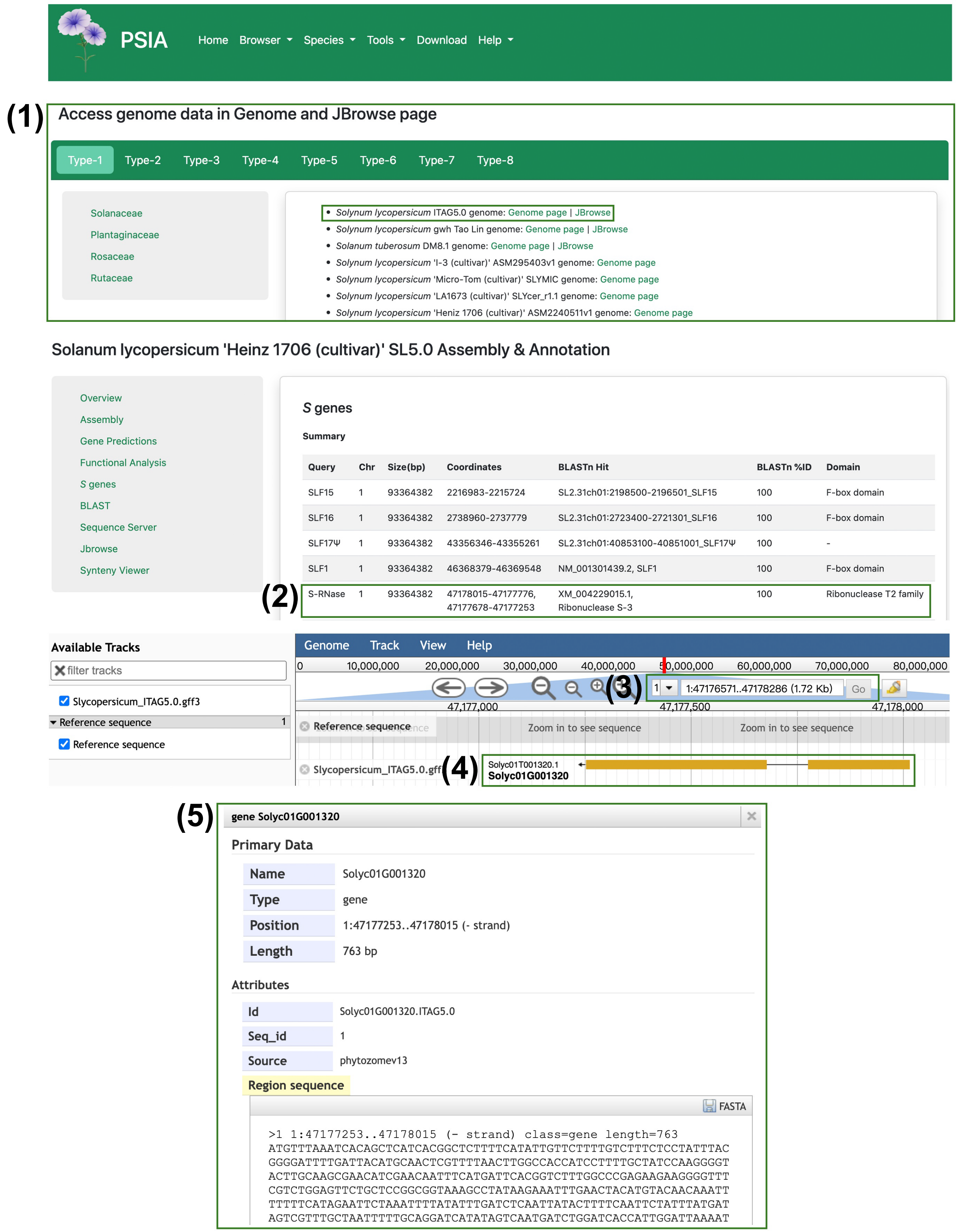
PSIA
Home
Browser
Species
Tools
Download
Help
(1)
Access genome data in Genome and JBrowse page
Type-1
Type-2
Type-3
Type-4
Type-5
Type-6
Type-7
Type-8
Solanaceae
Plantaginaceae
Rosaceae
Rutaceae
Solynum lycopersicum ITAG5.0 genome: Genome page | JBrowse
Solynum lycopersicum gwh Tao Lin genome: Genome page | JBrowse
Solanum tuberosum DM8.1 genome: Genome page | JBrowse
Solynum lycopersicum 'I-3 (cultivar)' ASM295403v1 genome: Genome page
Solynum lycopersicum 'Micro-Tom (cultivar)' SLYMIC genome: Genome page
Solynum lycopersicum 'LA1673 (cultivar)' SLYcer_r1.1 genome: Genome page
Solynum lycopersicum 'Heniz 1706 (cultivar)' ASM2240511v1 genome: Genome page
Solanum lycopersicum 'Heinz 1706 (cultivar)' SL5.0 Assembly & Annotation
Overview
Assembly
Gene Predictions
Functional Analysis
S genes
BLAST
Sequence Server
Jbrowse
Synteny Viewer
S genes
Summary
Query | Chr | Size(bp) | Coordinates | BLASTn Hit | BLASTn %ID | Domain
SLF15 | 1 | 93364382 | 2216983-2215724 | SL2.31ch01:2198500-2196501_SLF15 | 100 | F-box domain
SLF16 | 1 | 93364382 | 2738960-2737779 | SL2.31ch01:2723400-2721301_SLF16 | 100 | F-box domain
SLF17Ψ | 1 | 93364382 | 43356346-43355261 | SL2.31ch01:40853100-40851001_SLF17Ψ | 100 | -
SLF1 | 1 | 93364382 | 46368379-46369548 | NM_001301439.2, SLF1 | 100 | F-box domain
(2)
S-RNase | 1 | 93364382 | 47178015-47177776, 47177678-47177253 | XM_004229015.1, Ribonuclease S-3 | 100 | Ribonuclease T2 family
Available Tracks
filter tracks
Slycopersicum_ITAG5.0.gff3
Reference sequence
Reference sequence
Genome
Track
View
Help
0
10,000,000
20,000,000
30,000,000
40,000,000
50,000,000
60,000,000
70,000,000
80,000,000
(3)
1
1:47176571..47178286 (1.72 Kb)
Go
47,177,000
47,177,500
47,178,000
Reference sequence
Zoom in to see sequence
Zoom in to see sequence
Slycopersicum_ITAG5.0.gff
(4)
Solyc01T001320.1
Solyc01G001320
(5)
gene Solyc01G001320
Primary Data
Name
Solyc01G001320
Type
gene
Position
1:47177253..47178015 (- strand)
Length
763 bp
Attributes
Id
Solyc01G001320.ITAG5.0
Seq_id
1
Source
phytozomev13
Region sequence
FASTA
>1 1:47177253..47178015 (- strand) class=gene length=763
ATGTTTAAATCACAGCTCATCACGGCTCTTTTCATATTGTTCTTTTGTCTTTCTCCTATTTAC
GGGGATTTTGATTACATGCAACTCGTTTTAACTTGGCCACCATCCTTTTGCTATCCAAGGGGT
ACTTGCAAGCGAACATCGAACAATTTCATGATTCACGGTCTTTGGCCCGAGAAGAAGGGGTTT
CGTCTGGAGTTCTGCTCCGGCGGTAAAGCCTATAAGAAATTTGAACTACATGTACAACAAATT
TTTTTCATAGAATTCTAAATTTTATATTTGATCTCAATTATACTTTTCAATTCTATTTATGAT
AGTCGTTTGCTAATTTTTGCAGGATCATATAGTCAATGATCTGGATCACCATTGGATTAAAAT
