# Supplementary material for: PSIA: A Comprehensive Knowledgebase of Plant Self-incompatibility
Source: Genomics Proteomics Bioinformatics. 2025 May 21;23(3):qzaf046. doi: 10.1093/gpbjnl/qzaf046 (PMC12396629; doi:10.1093/gpbjnl/qzaf046)
Supplement: qzaf046_Supplementary_Data [file qzaf046_supplementary_data.zip › FigureS15.pdf]

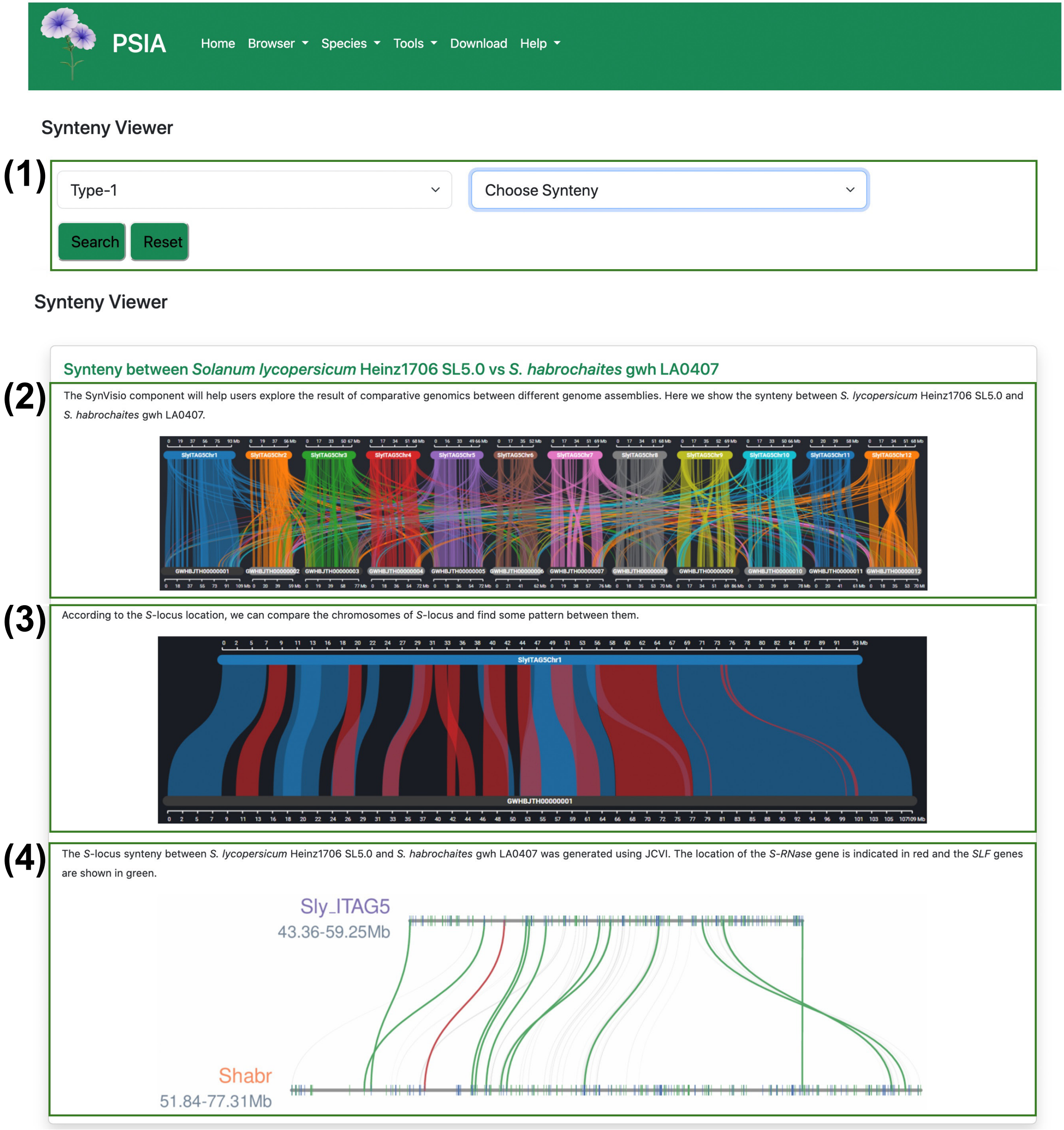
PSIA
Home
Browser
Species
Tools
Download
Help
Synteny Viewer
(1)
Type-1
Choose Synteny
Search
Reset
Synteny Viewer
Synteny between Solanum lycopersicum Heinz1706 SL5.0 vs S. habrochaites gwh LA0407
(2)
The SynVisio component will help users explore the result of comparative genomics between different genome assemblies. Here we show the synteny between S. lycopersicum Heinz1706 SL5.0 and S. habrochaites gwh LA0407.
(3)
According to the S-locus location, we can compare the chromosomes of S-locus and find some pattern between them.
SlyITAG5Chr1
GWHBJTH00000001
(4)
The S-locus synteny between S. lycopersicum Heinz1706 SL5.0 and S. habrochaites gwh LA0407 was generated using JCVI. The location of the S-RNase gene is indicated in red and the SLF genes are shown in green.
Sly_ITAG5
43.36-59.25Mb
Shabr
51.84-77.31Mb
