# Supplementary material for: PSIA: A Comprehensive Knowledgebase of Plant Self-incompatibility
Source: Genomics Proteomics Bioinformatics. 2025 May 21;23(3):qzaf046. doi: 10.1093/gpbjnl/qzaf046 (PMC12396629; doi:10.1093/gpbjnl/qzaf046)
Supplement: qzaf046_Supplementary_Data [file qzaf046_supplementary_data.zip › FigureS17.pdf]

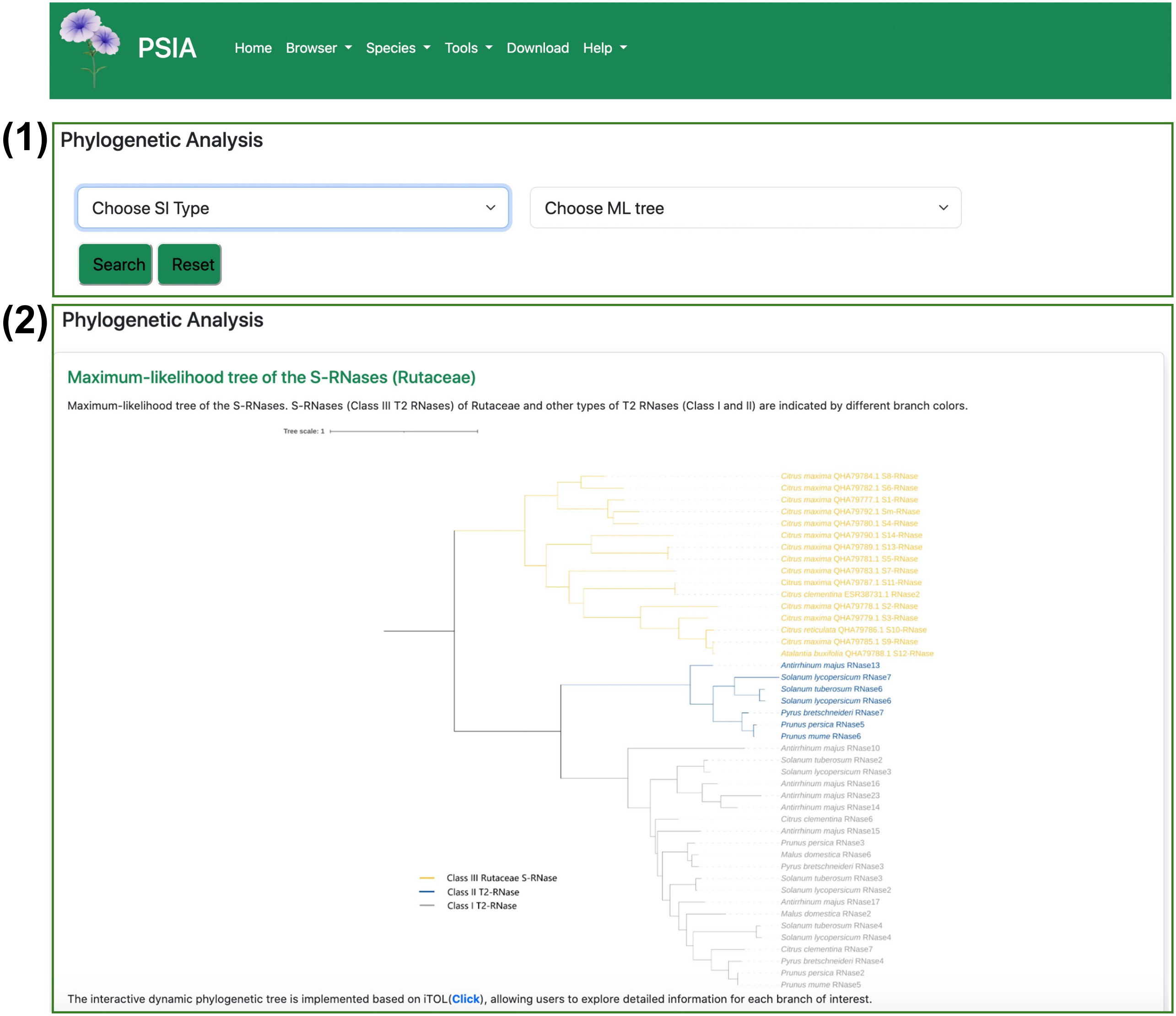
PSIA
Home
Browser
Species
Tools
Download
Help
(1)
Phylogenetic Analysis
Choose SI Type
Choose ML tree
Search
Reset
(2)
Phylogenetic Analysis
Maximum-likelihood tree of the S-RNases (Rutaceae)
Maximum-likelihood tree of the S-RNases. S-RNases (Class III T2 RNases) of Rutaceae and other types of T2 RNases (Class I and II) are indicated by different branch colors.
Tree scale: 1
Citrus maxima QHA79784.1 S8-RNase
Citrus maxima QHA79782.1 S6-RNase
Citrus maxima QHA79777.1 S1-RNase
Citrus maxima QHA79792.1 Sm-RNase
Citrus maxima QHA79780.1 S4-RNase
Citrus maxima QHA79790.1 S14-RNase
Citrus maxima QHA79789.1 S13-RNase
Citrus maxima QHA79781.1 S5-RNase
Citrus maxima QHA79783.1 S7-RNase
Citrus maxima QHA79787.1 S11-RNase
Citrus clementina ESR38731.1 RNase2
Citrus maxima QHA79778.1 S2-RNase
Citrus maxima QHA79779.1 S3-RNase
Citrus reticulata QHA79786.1 S10-RNase
Citrus maxima QHA79785.1 S9-RNase
Atalantia buxifolia QHA79788.1 S12-RNase
Antirrhinum majus RNase13
Solanum lycopersicum RNase7
Solanum tuberosum RNase6
Solanum lycopersicum RNase6
Pyrus bretschneideri RNase7
Prunus persica RNase5
Prunus mume RNase6
Antirrhinum majus RNase10
Solanum tuberosum RNase2
Solanum lycopersicum RNase3
Antirrhinum majus RNase16
Antirrhinum majus RNase23
Antirrhinum majus RNase14
Citrus clementina RNase6
Antirrhinum majus RNase15
Prunus persica RNase3
Malus domestica RNase6
Pyrus bretschneideri RNase3
Solanum tuberosum RNase3
Solanum lycopersicum RNase2
Antirrhinum majus RNase17
Malus domestica RNase2
Solanum tuberosum RNase4
Solanum lycopersicum RNase4
Citrus clementina RNase7
Pyrus bretschneideri RNase4
Prunus persica RNase2
Prunus mume RNase5
Class III Rutaceae S-RNase
Class II T2-RNase
Class I T2-RNase
The interactive dynamic phylogenetic tree is implemented based on iTOL(Click), allowing users to explore detailed information for each branch of interest.
