# Supplementary material for: PSIA: A Comprehensive Knowledgebase of Plant Self-incompatibility
Source: Genomics Proteomics Bioinformatics. 2025 May 21;23(3):qzaf046. doi: 10.1093/gpbjnl/qzaf046 (PMC12396629; doi:10.1093/gpbjnl/qzaf046)
Supplement: qzaf046_Supplementary_Data [file qzaf046_supplementary_data.zip › FigureS19.pdf]

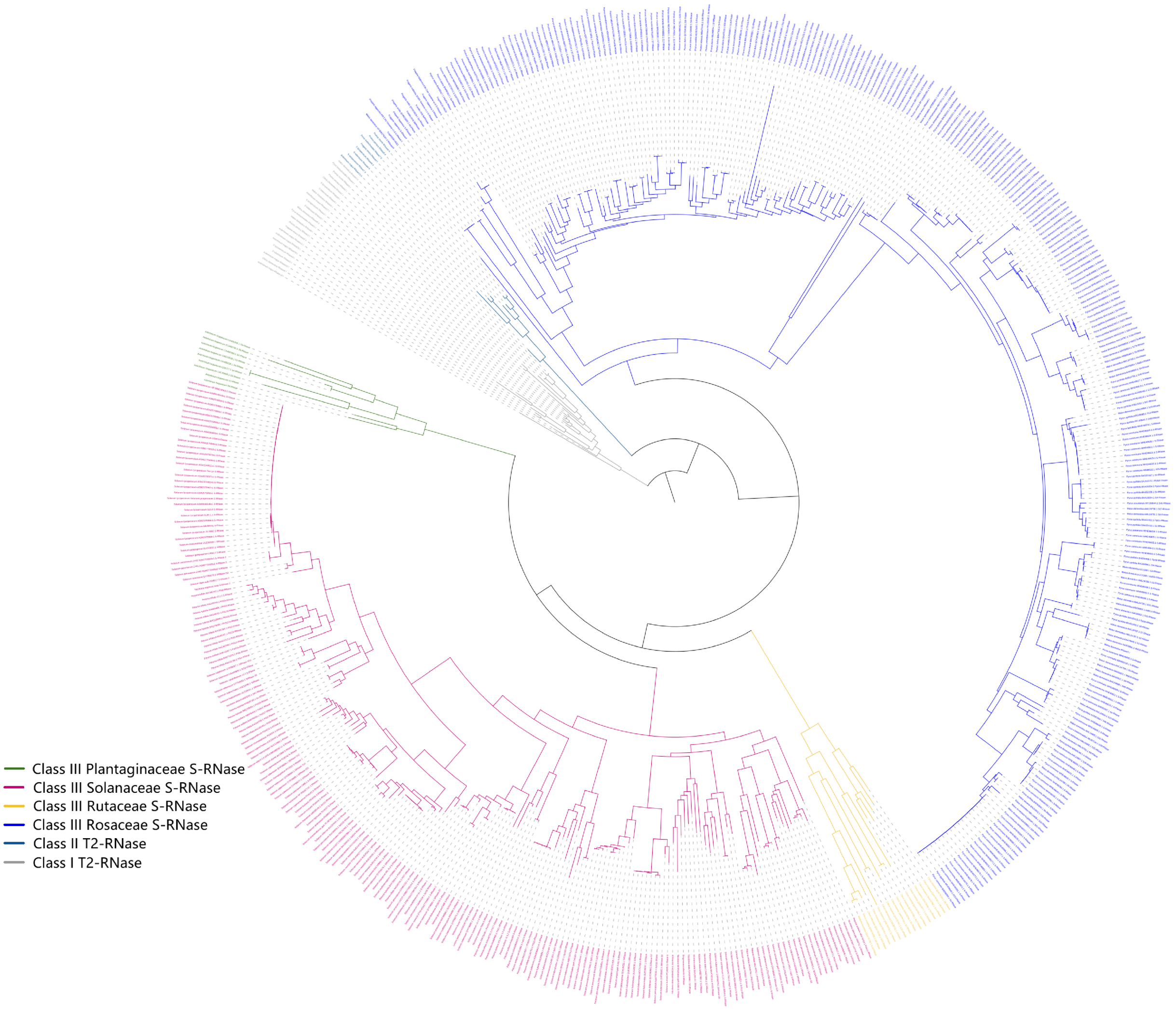
Class III Plantaginaceae S-RNase
Class III Solanaceae S-RNase
Class III Rutaceae S-RNase
Class III Rosaceae S-RNase
Class II T2-RNase
Class I T2-RNase
