# Supplementary material for: PSIA: A Comprehensive Knowledgebase of Plant Self-incompatibility
Source: Genomics Proteomics Bioinformatics. 2025 May 21;23(3):qzaf046. doi: 10.1093/gpbjnl/qzaf046 (PMC12396629; doi:10.1093/gpbjnl/qzaf046)
Supplement: qzaf046_Supplementary_Data [file qzaf046_supplementary_data.zip › FigureS20.pdf]

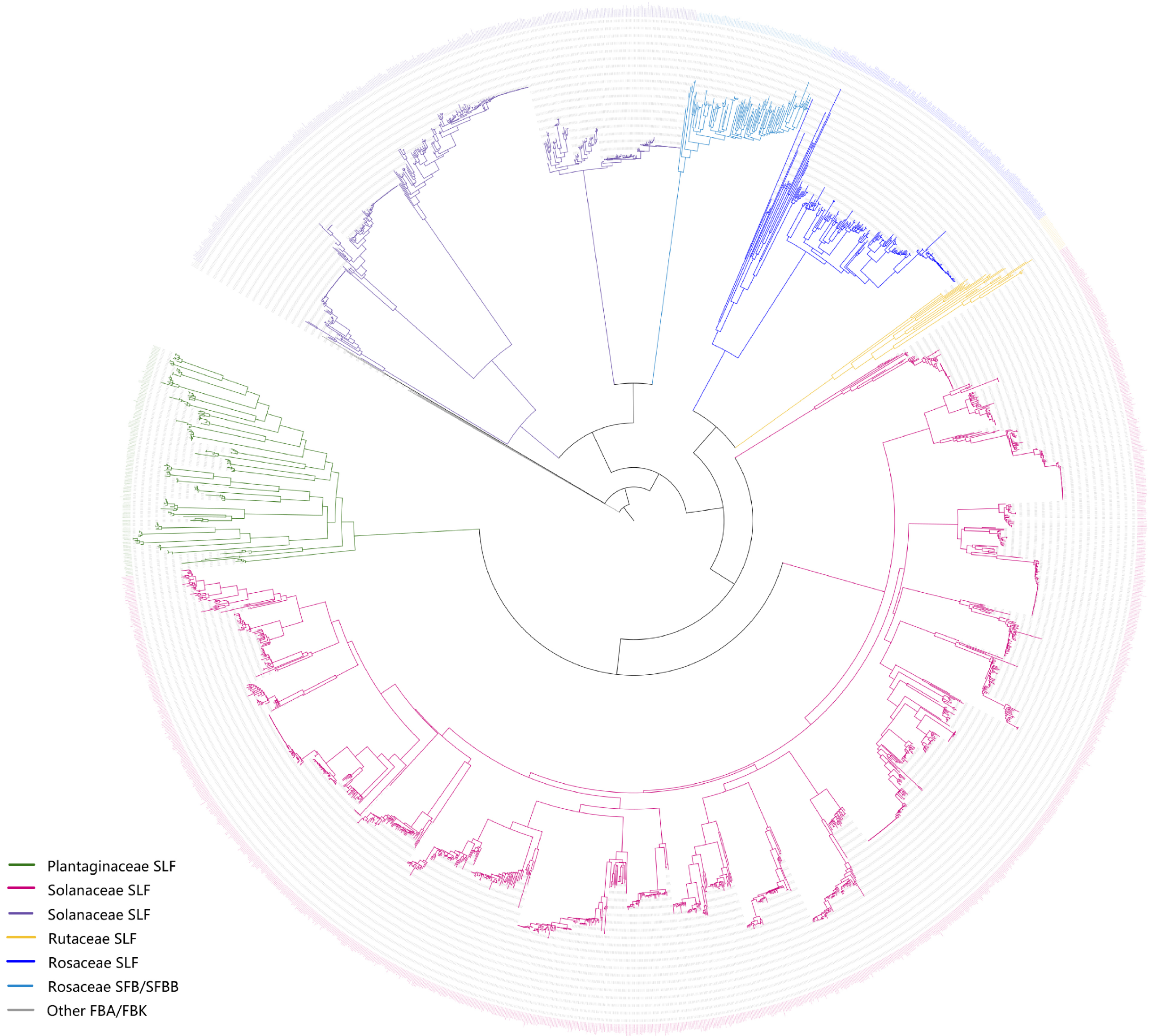

Plantaginaceae SLF
Solanaceae SLF
Solanaceae SLF
Rutaceae SLF
Rosaceae SLF
Rosaceae SFB/SFBB
Other FBA/FBK
