# Supplementary material for: PSIA: A Comprehensive Knowledgebase of Plant Self-incompatibility
Source: Genomics Proteomics Bioinformatics. 2025 May 21;23(3):qzaf046. doi: 10.1093/gpbjnl/qzaf046 (PMC12396629; doi:10.1093/gpbjnl/qzaf046)
Supplement: qzaf046_Supplementary_Data [file qzaf046_supplementary_data.zip › Supplementary material captions.docx]

**Supplementary materials**

**Figure S1 Number of assembled species corresponding to the eight types of SI plants**

**Figure S2 Species tree of the eight types of SI plants included in PSIA**

The species tree was constructed using the TimeTree database and annotated with iTOL. Species from 11 families are highlighted in distinct colors.

**Figure S3 The phylogenetic tree of species at the family level showing the origin of the eight SI types**

The evolutionary tree is generated by TimeTree. The geological time scale is shown at the top, while the bottom numerical axis represents the evolutionary timeline. The circles of different colors and their corresponding lines represent the eight types of SI. The orange dashed line indicates the ancient type-1 *S* structure.

**Figure S4 Molecular mechanism of type-1 SI**

The system with the broadest taxonomic distribution, which we term type-1 SI, is gametophytic SI and based on linked pistil *S-RNase* and pollen *S-locus F-box* (*SLF*)/*S-haplotype-specific F-box* (*SFB*). So far, type-1 SI has been found in four eudicot families: Solanaceae, Plantaginaceae, Rosaceae, and Rutaceae. After pollination, both S_1_- and S_2_-RNases can enter pollen tubes and be recognized by S_1_- or S_3_-SLFs, but only SCF^S3-SLF^ complexes can ubiquitinate these S-RNases leading to their degradation by 26S proteasome, with the survived S_1_-RNase in *S_1_* pollen tubes forming the S-RNase condensates (SRCs) resulting in self-pollen inhibition.

**Figure S5 Molecular mechanism of type-2 SI**

Type-2 SI is the sporophytic Brassicaceae-type SI, controlled by a male *S*-locus cysteine-rich (SCR) protein/*S*-locus protein 11 and a female *S*-locus receptor kinase (SRK). Pollen S_1_-SCR can specifically recognize its cognate S_1_-SRK after pollination, triggering several signaling cascades mediated by phosphorylation and ubiquitin-proteasome system, thus leading to self-pollen rejection.

**Figure S6 Molecular mechanism of type-3 SI**

Type-3 is the gametophytic Papaveraceae-type SI, possessing the common poppy (*Papaver rhoeas*) stigma *S* (PrsS) and *P. rhoeas* pollen *S* (PrpS). Prs S_2_ secreted by *S_1_S_2_* papilla cells can specifically bind to pollen membrane-localized Prp S_2_, stimulating Ca^2+^ influx, ROS accumulation, actin depolymerization, and PCD of self-pollen.

**Figure S7 Molecular mechanism of type-4/5 SI**

Type-4 and -5 are the sporophytic heterostyly SI of Primulaceae and Turneraceae, controlled by hemizygous *S*-loci mainly encoding *CYP* (Cytochrome P450) and *BAHD*, respectively. Although both can inactivate pistil BR, they are absent in long styles, resulting in non-self-pollen acceptance and self-pollen rejection.

**Figure S8 Molecular mechanism of type-6 SI**

We classified Poaceae SI as type-6, gametophytically controlled by two multiallelic and independent loci, *S* and *Z*. Taking *S* as an example, stamen-expressed transmembrane polypeptides containing a domain of unknown function 247 (DUF247) can specifically recognize the pistil-specific peptide HPS10, resulting in the rejection of the self-pollen.

**Figure S9 Molecular mechanism of type-7 SI**

We classified Linaceae heterostyly SI as type-7, governed by two distyly candidate genes (*TSS1* and *WDR-44*) expressed exclusively by the dominant *S* allele, which regulate cell expression, BR, and auxin responses in short styles.

**Figure S10 Molecular mechanism of type-8 SI**

We classified Oleaceae SI as type-8. The presence/absence polymorphism of the *GA2ox-S* gene is stably associated with SI groups across Oleaceae. GA3 treatment can switch the female specificity of [Hb] individuals and the male specificity of [Ha] ones.

**Figure S11 Web interface of PSIA**

**A.** Navigation bar of PSIA. The "Browser" section provides access to genome information, SI types, and SI gene introductions. The "Species" section includes representative species of the eight SI types. The "Tools" section offers several utility tools available in PSIA. A fuzzy search function is integrated on the right side. **B.** Overview of PSIA, comprising three sections: brief information about PSIA; release notes for genome assemblies; and news and updates related to PSIA. **C.** The "Knowledge" module of PSIA, featuring three components: the origin and evolution of the eight SI types; eight diagrams illustrating different molecular mechanisms; and two reported working models of SI identified in the representative species *Petunia hybrida*. **D.** Quick-access links for 11 SI families. **E.** Phylogenetic positions of SI plants with sequenced and published genomes.

**Figure S12 Workflow and results of BLAST in PSIA**

ViroBLAST has been integrated into PSIA, enhancing its BLAST functionality for genome-wide searches of self-incompatibility (SI) species. (1) Users can input a DNA or protein sequence. (2) Select the “blastn” program for DNA sequences or “blastp” for protein sequences. (3) Choose the appropriate BLAST local database for an SI species. (4) Obtain results using default parameters by clicking “Basic Search”. In most cases, the default parameters in “Basic Search” yield optimal results. (5) For users requiring parameter customization, the “Advanced Search” option allows fine-tuning of search settings before execution. (6) The BLAST results interface displays all matched alignments sorted by score. Results can be filtered based on similarity percentage or BLAST score.

**Figure S13 Workflow and results of SequenceServer** **in PSIA**

SequenceServer has been integrated into PSIA, and local BLAST databases have been constructed using thousands of *S* gene sequences, including publicly known and newly identified sequences. (1) Users can input a DNA or protein sequence. (2) Select the appropriate BLAST local database of *S* genes. (3–4) Obtain results using default parameters. (5) The results page displays a graphical overview and a summary table of hits.

**Figure S14 Example of JBrowse usage in PSIA**

Genome assemblies with available GFF files provide JBrowse functionality, enabling researchers to explore the entire genome and *S*-locus. An example of JBrowse usage is as follows: (1) Select the JBrowse link for *Solanum lycopersicum* SL5.0. (2) View *S-RNase* gene information, including chromosomal location and genomic coordinates within the genome assembly. (3) In the JBrowse interface, enter the chromosome and genomic coordinates to visualize the gene structure of the *S-RNase* gene. (4–5) Click on the *S-RNase* gene to display detailed information about its structure and annotations.

**Figure S15 Workflow and example analysis results of Synteny Viewer**

(1) In the Synteny Viewer tool (accessed via the navigation bar), select the SI type-1 option in the search interface and choose ‘*Solanum lycopersicum* Heinz1706 SL5.0 vs. *S. habrochaites* gwh LA0407’ from the right-side dropdown menu. (2) The output displays chromosomal syntenic relationship between *S. lycopersicum* Heinz1706 SL5.0 and *S. habrochaites* gwh LA0407. (3) The synteny between the chromosomes of the *S*-locus is visualized. (4) JCVI-generated *S*-locus synteny comparison between *S. lycopersicum* Heinz1706 SL5.0 and *S. habrochaites* gwh LA0407, highlighting the *S-RNase* gene (red) and *SLF* genes (green).

**Figure S16 Comparative synteny analysis of the *S*-locus across *Solanum* species**

Syntenic relationships were analyzed using JCVI, with *S-RNase* genes highlighted in red and *SLF* genes in green.

**Figure S17 Workflow and example analysis results of Phylogenetic Analysis**

(1) Using the Phylogenetic Analysis tool (accessed via the navigation bar), select the SI type-1 option in the search interface and choose “ML tree of S-RNases (Rutaceae)” from the right-side dropdown menu. (2) The resulting phylogenetic tree displays evolutionary relationships among S-RNases (Class III T2 RNases) from Rutaceae and other T2 RNase classes (I and II).

**Figure S18 Maximum-likelihood phylogenetic tree of *S-locus* *F-box* (*SLF*) genes from Solanaceae**

The tree includes comparative analysis with other F-box proteins (FBA/FBKs), with distinct branch colors representing different clusters.

**Figure S19 Maximum-likelihood tree of S-RNases from four type-1 SI families**

The maximum-likelihood tree shows evolutionary relationships among S-RNases (Class III T2 RNases) from Plantaginaceae, Solanaceae, Rutaceae, and Rosaceae, with Class I and II T2 RNases included as outgroups. Distinct branch colors represent different clusters.

**Figure S20 Maximum-likelihood tree of the SLFs/SFBs/SFBBs of four type-1 SI families**

The maximum-likelihood tree illustrates evolutionary divergence among SLFs/SFBs/SFBBs proteins from Plantaginaceae, Solanaceae, Rutaceae, and Rosaceae, with related F-box proteins (FBA/FBKs) included as outgroups. Distinct branch colors represent different clusters.
