# Supplementary figures and images for: PSIA: A Comprehensive Knowledgebase of Plant Self-incompatibility
Source: Genomics Proteomics Bioinformatics. 2025 May 21;23(3):qzaf046. doi: 10.1093/gpbjnl/qzaf046 (PMC12396629; doi:10.1093/gpbjnl/qzaf046)

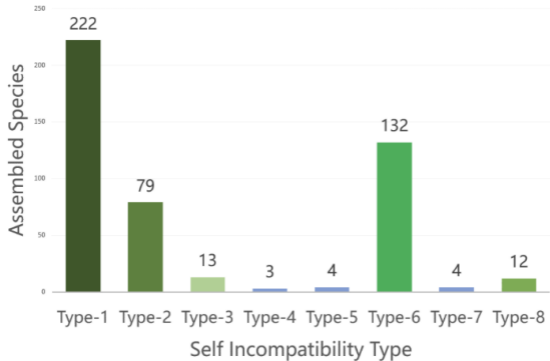

Supplement: qzaf046_Supplementary_Data [file qzaf046_supplementary_data.zip › FigureS1.pdf]

# Type-8 SI

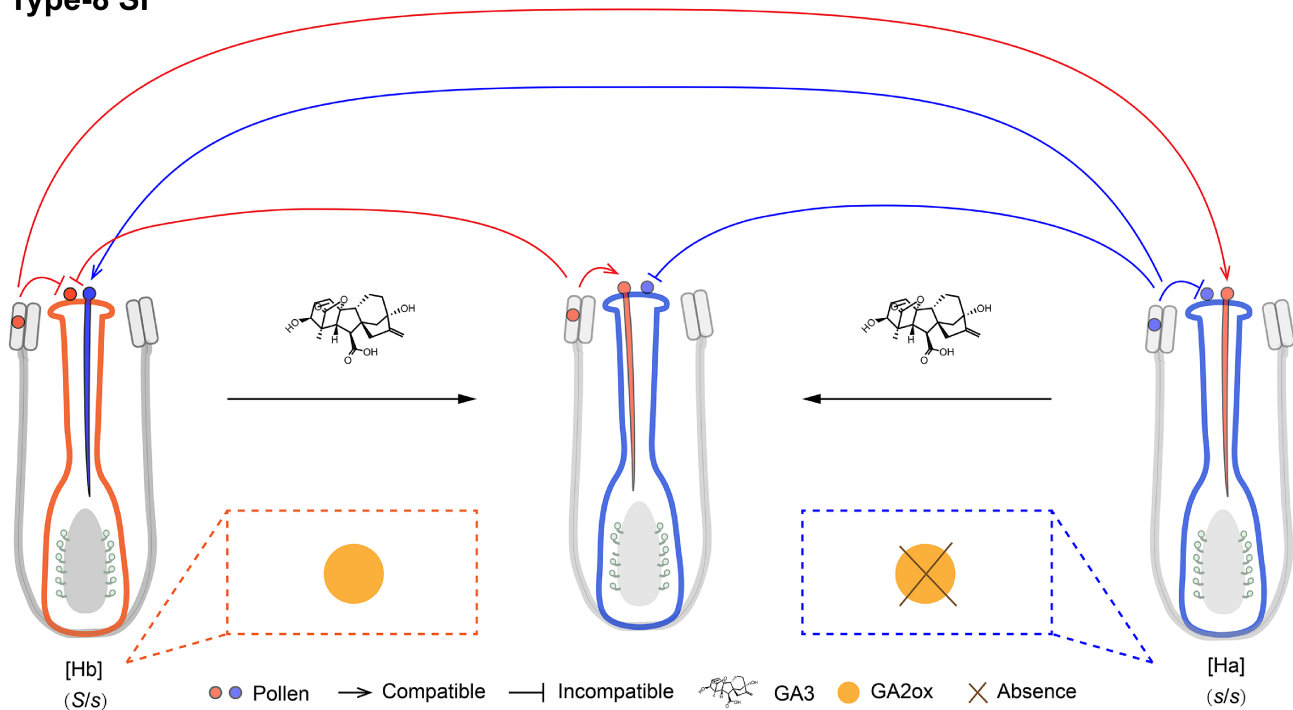

Supplement: qzaf046_Supplementary_Data [file qzaf046_supplementary_data.zip › FigureS10.pdf]

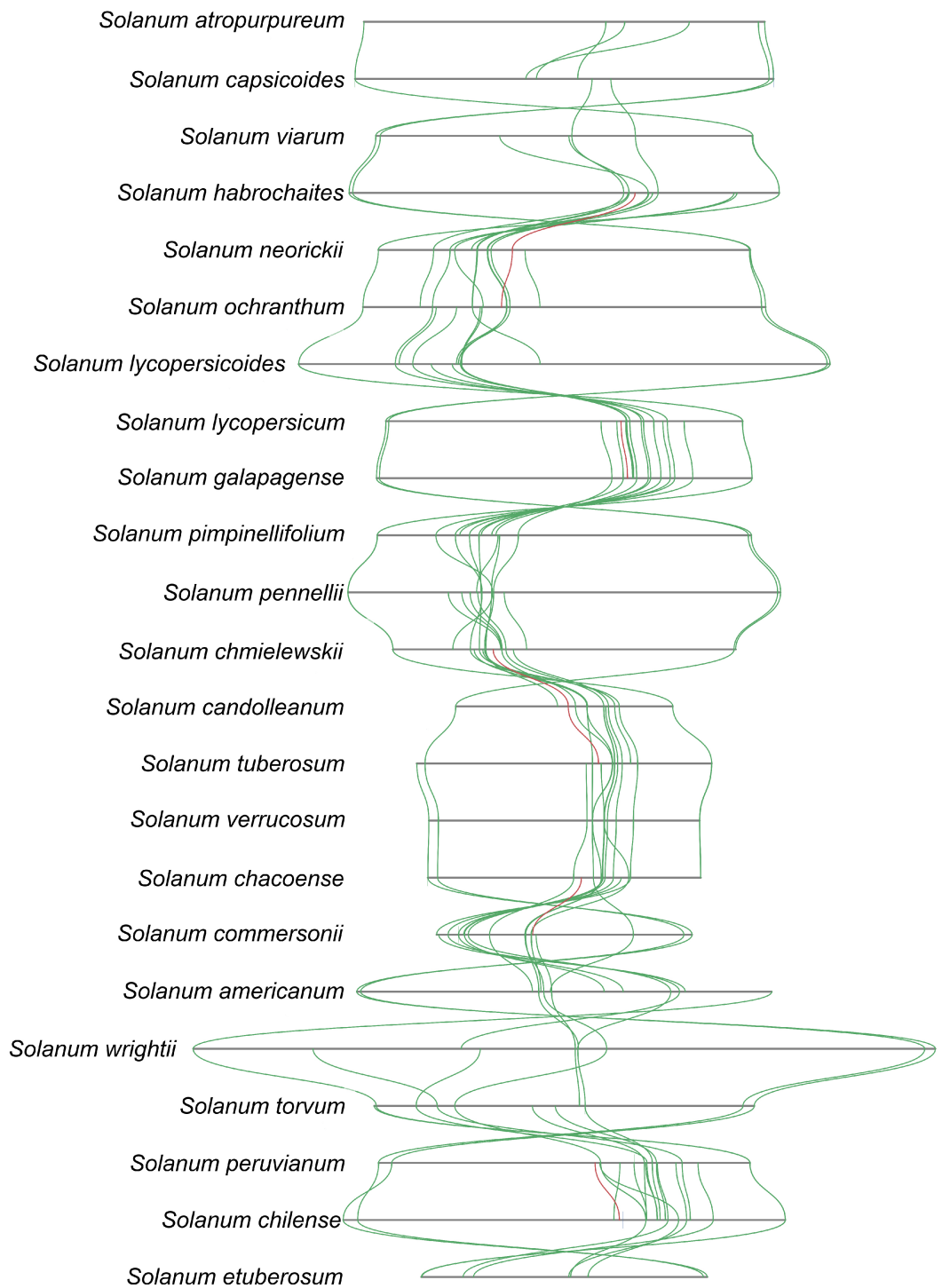

Supplement: qzaf046_Supplementary_Data [file qzaf046_supplementary_data.zip › FigureS16.pdf]

— Solanaceae SLF  
— Other FBA/FBK

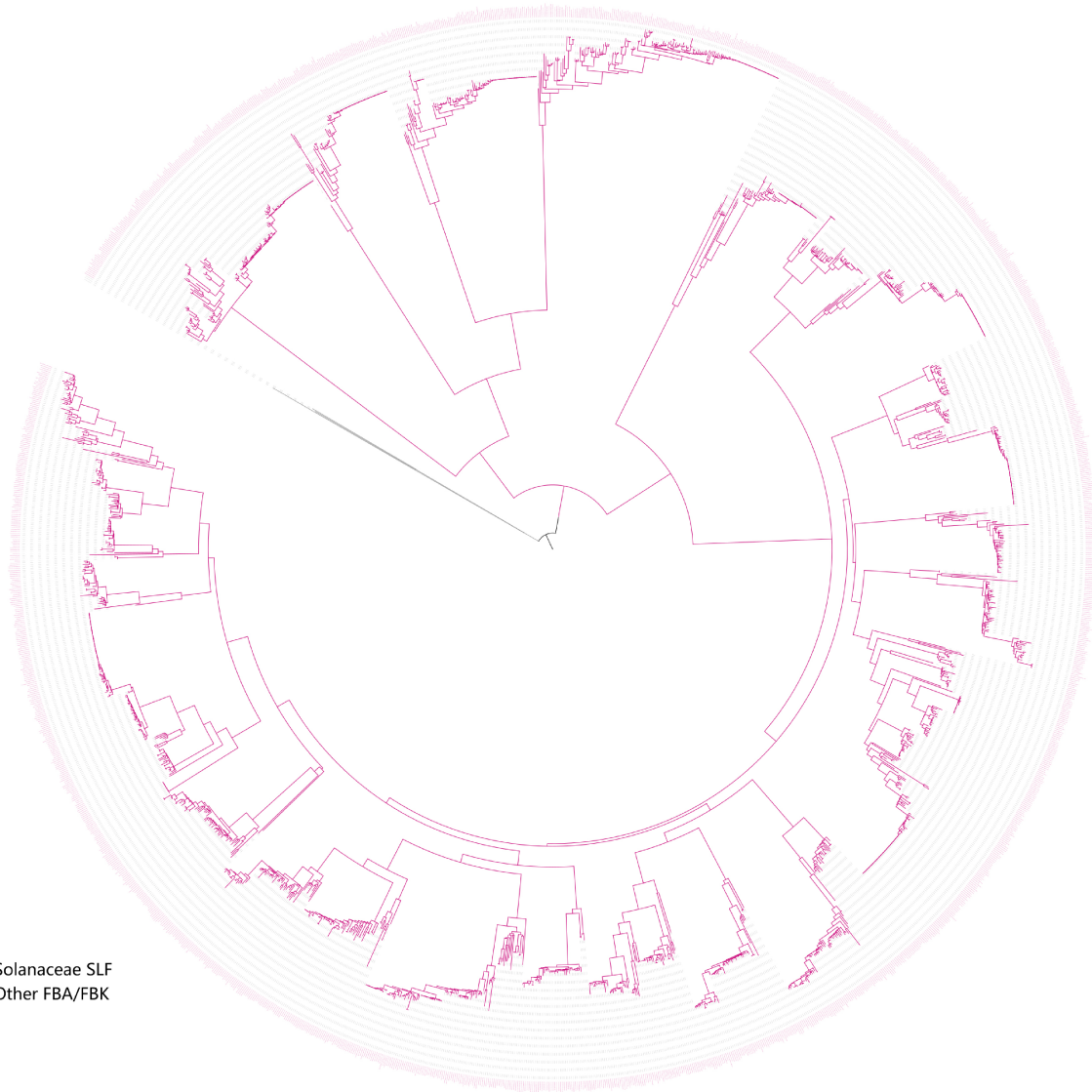

Supplement: qzaf046_Supplementary_Data [file qzaf046_supplementary_data.zip › FigureS18.pdf]

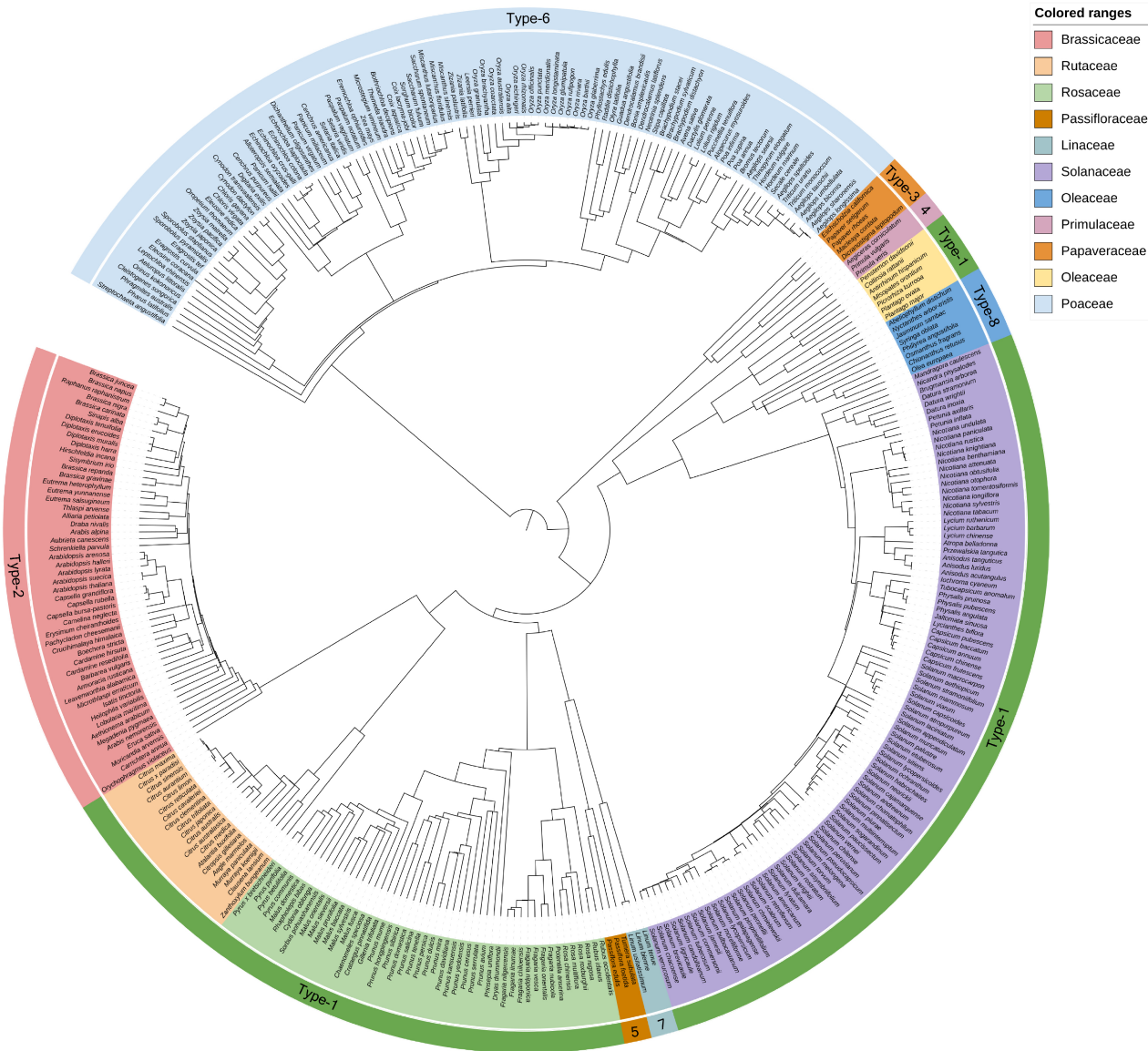

Supplement: qzaf046_Supplementary_Data [file qzaf046_supplementary_data.zip › FigureS2.pdf]

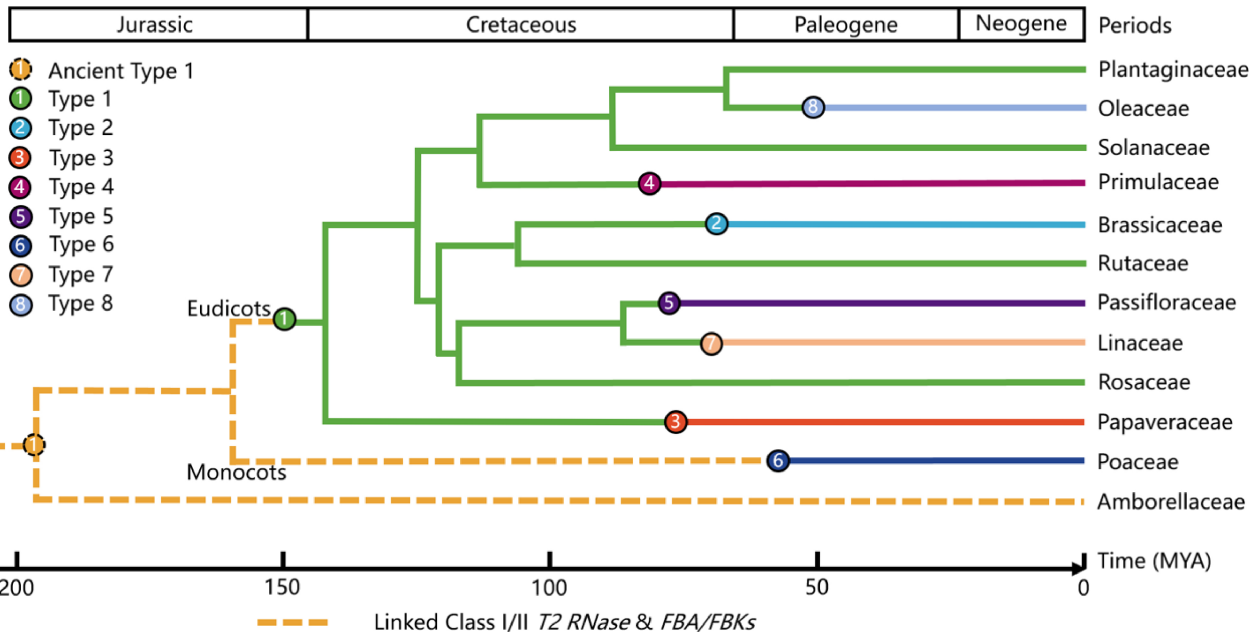

Supplement: qzaf046_Supplementary_Data [file qzaf046_supplementary_data.zip › FigureS3.pdf]

# Type-1 SI

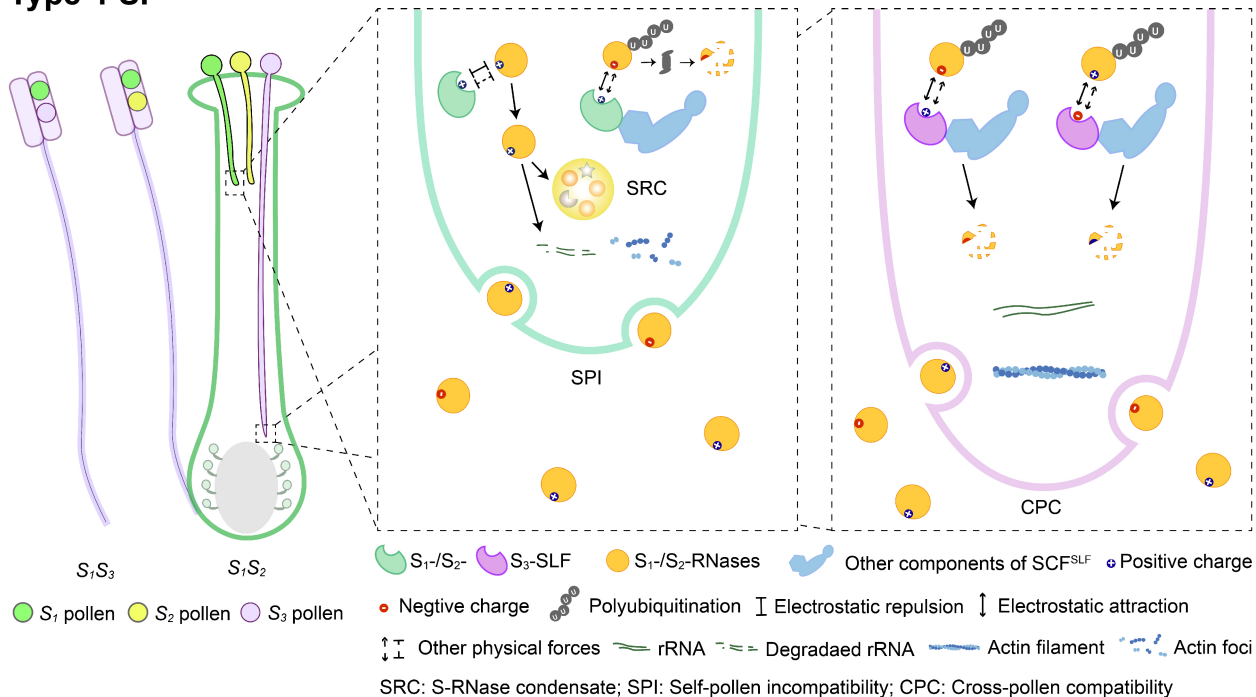

Supplement: qzaf046_Supplementary_Data [file qzaf046_supplementary_data.zip › FigureS4.pdf]

# Type-2 SI

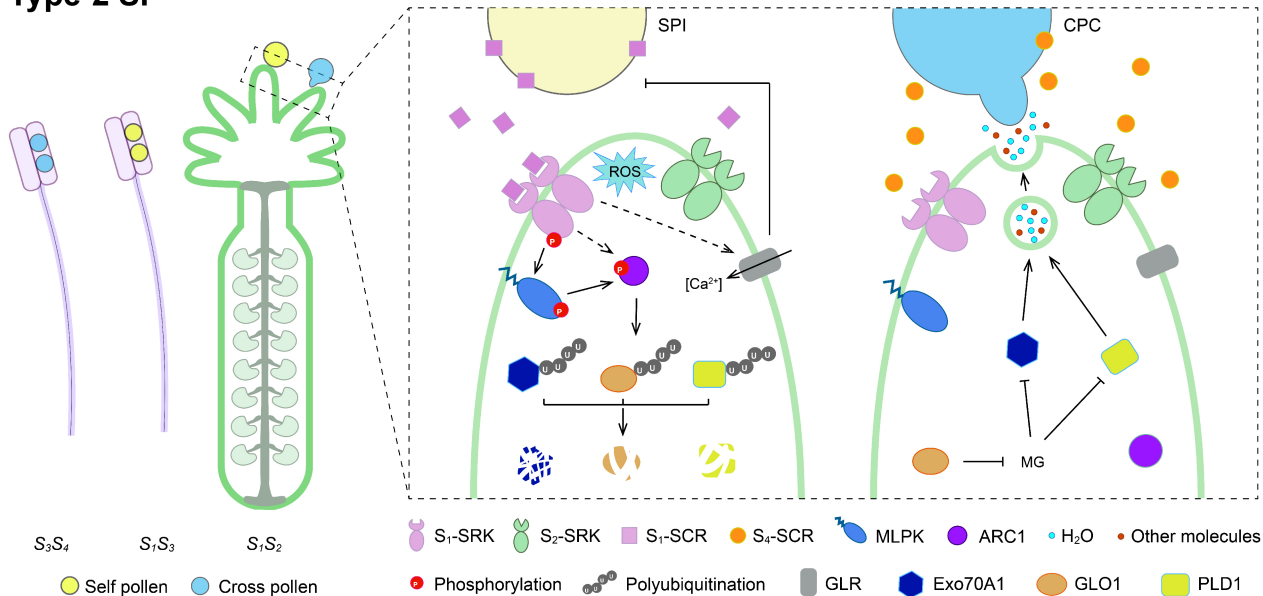

ROS: Reactive oxygen species; MG: Methylglyoxal

Supplement: qzaf046_Supplementary_Data [file qzaf046_supplementary_data.zip › FigureS5.pdf]

# Type-3 SI

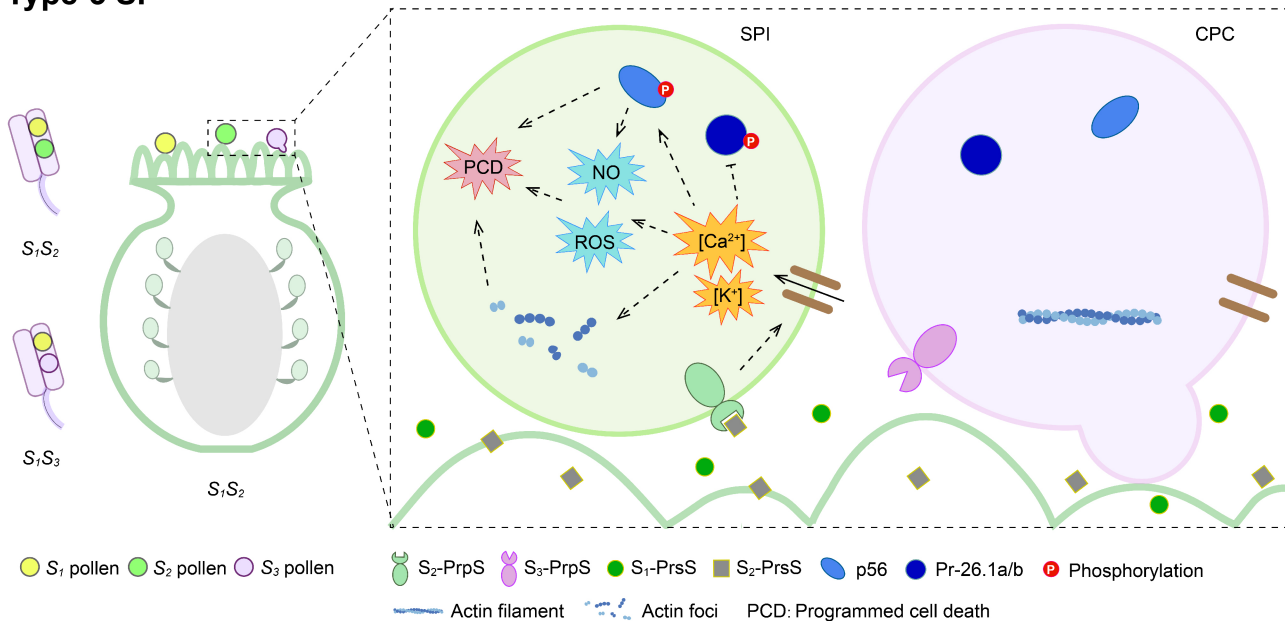

Supplement: qzaf046_Supplementary_Data [file qzaf046_supplementary_data.zip › FigureS6.pdf]

# Type-4/5 SI

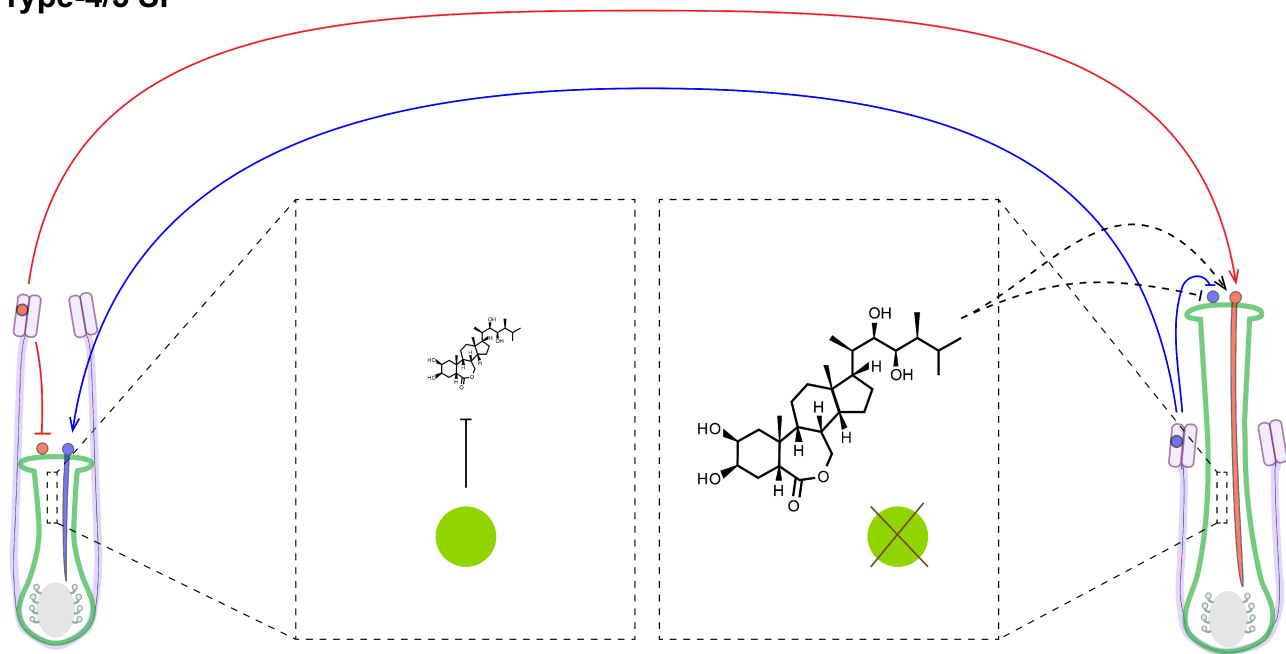

Thrum  
(S/s)

● ● Pollen

→ Compatible

—| Incompatible

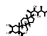

Brassinosteroid, BR

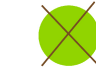

CYP734A50/BAHD

✗ Absence

Pin  
(s/s)

Supplement: qzaf046_Supplementary_Data [file qzaf046_supplementary_data.zip › FigureS7.pdf]

# Type-6 SI

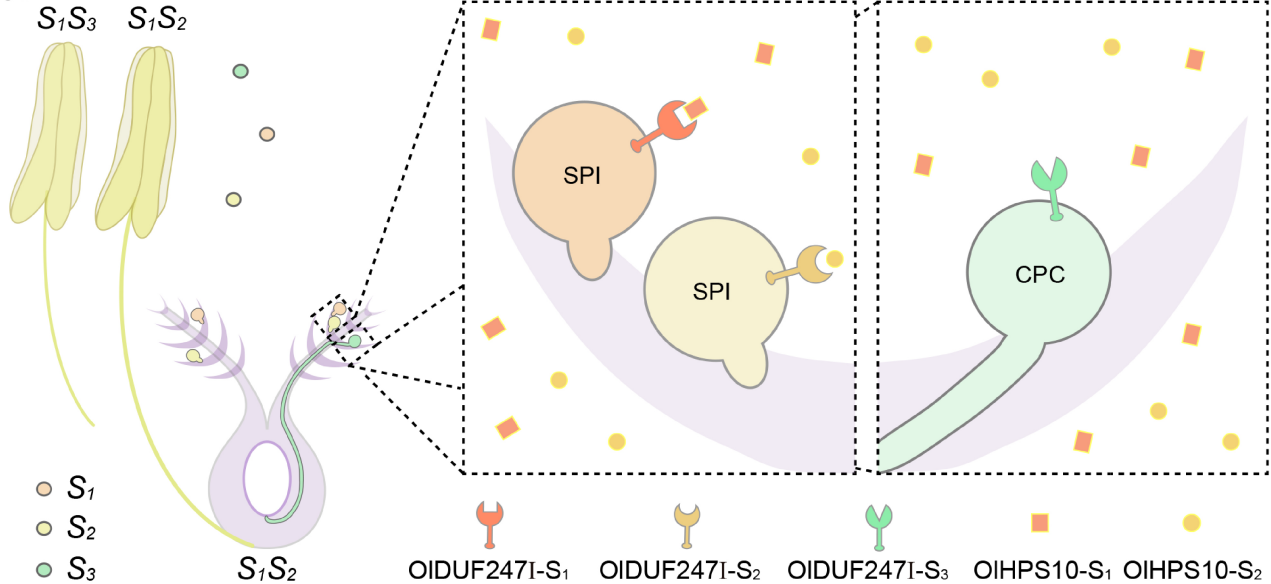

Supplement: qzaf046_Supplementary_Data [file qzaf046_supplementary_data.zip › FigureS8.pdf]

# Type-7 SI

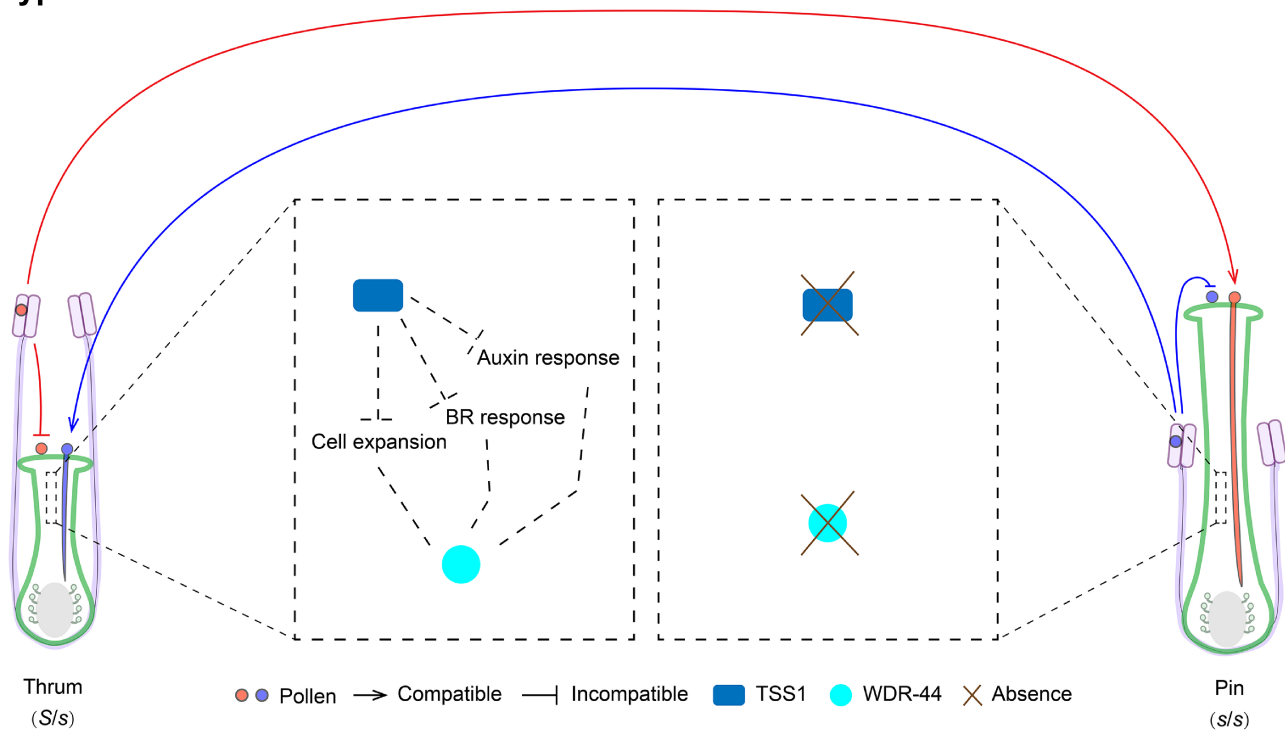

Supplement: qzaf046_Supplementary_Data [file qzaf046_supplementary_data.zip › FigureS9.pdf]
